# Interfacial Salinity–Transport Matching in 3D Solar Evaporators: A Framework for Brine‐Stable Solar Interfacial Evaporation

**DOI:** 10.1002/advs.77527

**Published:** 2026-08-30

**Authors:** Mohammad Sajad Sorayani Bafqi, Arun Prakash Aranga Raju, Omid Doustdar, Reza Nekouie Esfahani, Ali Sadaghiani

**Affiliations:** ^1^ SMaRT Research Group Department of Mechanical Engineering School of Engineering University of Birmingham Birmingham UK; ^2^ Levidian Nanosystems Limited Levidian Technology Centre Cambridge UK; ^3^ The Manufacturing Technology Centre Ltd Coventry UK

**Keywords:** 3D solar evaporator, brine evaporation, capillary replenishment, interfacial salinity, salt transport, solar‐driven interfacial evaporation, transport matching

## Abstract

Solar‐driven interfacial evaporation is commonly optimized through photothermal absorption and evaporation flux normalized by projected illuminated area. This metric is useful for quasi‐2D evaporators and dilute feeds, but it does not resolve the coupled constraints that emerge in 3D architectures during brine operation. Here, porous polyvinyl alcohol/graphene nanoplatelet evaporators are used to identify the transition from evaporation‐cooled operation to brine transport limitation. GNP incorporation increases dry photothermal temperatures to 150–180°C; however, this dry‐state ranking is not preserved under brine. Under DI water and 3.5 wt.% NaCl, evaporative cooling suppresses dry‐temperature differences and produces stable wet operation. Under 20 wt.% NaCl, several architectures develop delayed sensible heat accumulation after the early operating period despite no visible salt accumulation. Two evaporators with similar dry photothermal temperatures show sharply different brine responses: the low exposed‐boundary structure reaches 131.34°C after 4 h, whereas the high exposed‐boundary structure shows only 5.34°C late‐stage thermal drift and retains the highest brine‐flux stability. Local conductivity, salt mass balance, transport descriptors, and side‐sealed controls show that brine‐stable 3D evaporation requires matched heat generation, capillary replenishment, salt redistribution, and vapor removal, rather than maximum dry temperature, first‐hour flux, or visual salt suppression as sole performance criteria alone.

## Graphical abstract

A transport‐matching framework is developed to evaluate brine‐stable 3D solar evaporators beyond evaporation rate alone. Using PVA/GNP porous evaporators as a model system, the study shows that stable brine evaporation requires balanced heat generation, liquid replenishment, salt redistribution, and vapor removal rather than maximum dry temperature or visible salt suppression.

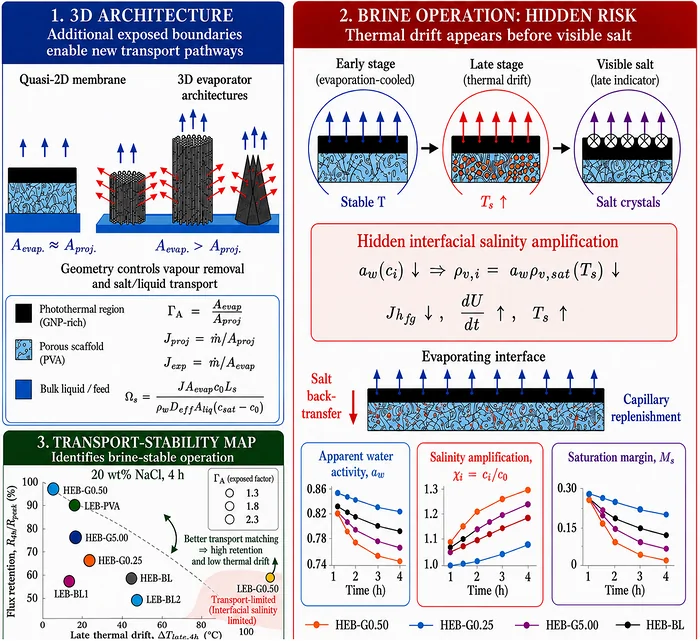

## 1 Introduction

Solar‐driven interfacial evaporation localizes solar heat at the liquid–vapor interface and reduces the thermal penalty associated with bulk‐water heating. The heat‐localization concept was established through structures that combine strong solar absorption, thermal insulation, hydrophilic water supply, and interconnected pores, allowing heat and liquid flow to converge at the evaporation region [1, 2]. Ghasemi et al. [3] demonstrated solar steam generation by heat localization and reported high solar thermal efficiency under concentrated irradiation. Subsequent work has expanded this principle into a broad class of solar absorbers, evaporation structures, thermal insulators, and thermal concentrators for desalination, wastewater treatment, and brine management [4, 5].

Evaporation flux remains the dominant performance metric in solar interfacial evaporation. Total mass loss is commonly normalized by the projected illuminated area, which is appropriate when evaporation is dominated by the top surface of a quasi‐2D membrane. In 3D evaporator architectures, however, the projected area no longer defines the complete evaporation boundary. Fins, pillars, bridges, lattices, folded surfaces, porous monoliths and open channels introduce additional exposed evaporation surfaces, non‐planar vapor‐removal pathways and architecture‐dependent liquid/salt transport routes. A 3D evaporator therefore changes the thermal boundary, vapor field, and salt/liquid transport pathway simultaneously.

This distinction becomes critical under brine operation. Under low salinity, increasing photothermal absorption or exposed evaporation boundaries can increase apparent vapor generation. Under brine conditions, water removal enriches salt near the evaporating interface and reduces water activity before visible crystallization appears [6, 7]. The resulting decrease in vapor‐pressure driving force weakens evaporative cooling and can increase sensible heat storage even when the photothermal layer remains highly absorptive. Brine stability therefore depends on whether evaporation‐driven salt loading is balanced by salt redistribution and capillary replenishment.

Leading studies on evaporators have addressed this challenge by introducing confined water layers [8, 9], mass‐transfer bridges [9, 10], function‐separated Janus structures [9, 11], salt‐localized crystallization [12, 13] and multistage architectures [14, 15]. More recently, regulating ion transport, concentration polarization, and salt redistribution within porous architectures has been recognized as an equally important strategy for maintaining stable evaporation under high‐salinity conditions [16, 17, 18]. These studies show that high‐salinity operation is not controlled by heat localization alone. For example, confined‐water‐layer evaporators can sustain high‐salinity operation when thermal localization and salt rejection are coupled [8]. 3D open architectures with mass‐transfer bridges show that insufficient liquid/salt transport leads to evaporation‐rate decline because the evaporation‐surface salinity increases [10]. Recent analyses of 3D evaporators further show that projected‐area flux can exceed the nominal one‐sun solar‐thermal limit when non‐projected sidewalls exchange heat with the environment and contribute to vapor release [19].

The unresolved design criterion is therefore not the absence of salt accumulation. Salt accumulation, interfacial salinity rise, and concentration polarization are intrinsic to brine evaporation. The critical transition occurs when heat generation exceeds the combined capacity for salt redistribution, liquid replenishment, and vapor removal. At that point, the evaporator shifts from evaporation‐cooled operation to heat‐accumulation‐dominated operation. A structure that performs well under DI water or seawater‐level salinity can become unstable under brine if the same architecture amplifies interfacial salinity or restricts liquid supply.

Porous PVA/GNP evaporators with controlled photothermal loading, exposed‐boundary factor, and bilayer architecture provide a model system for resolving this transition. Dry photothermal tests quantify heat‐generation capacity. DI water and 3.5 wt.% NaCl tests establish evaporation‐cooled behavior under low and moderate salinity. A 20 wt.% NaCl brine‐stress test separates early photothermal activation from delayed salt/liquid‐transport limitation. Local conductivity measurements and salt mass balance before and after operation quantify architecture‐dependent interfacial salt enrichment. Effective salt diffusivity, liquid/salt transport cross‐section, and salt‐transport length are measured independently and used to evaluate a salt‐loading balance. The measured thermal drift, flux retention, projected/exposed‐boundary flux, side‐sealed controls, and energy consistency are then combined into an interfacial salinity–transport matching criterion for 3D brine evaporators.

## 2 Results and Discussion

### 2.1 Sample Coding and Exposed‐Boundary Classes

The sample codes used in this study follow the exposed‐boundary factor, Γ_*A*_ =  *A_evap_*/*A_proj_*, where *A_evap_* is the exposed evaporative boundary and *A_proj_* is the projected illuminated area. LEB denotes the low exposed‐boundary class, HEB symbolizes the high exposed‐boundary class, G0.25, G0.50, and G5.00 represent GNP loading, and BL denotes bilayer architecture. The LEB and HEB groups were designed as representative exposed‐boundary architectural classes based on a simplified cylindrical evaporator model. These classes provide a model platform for evaluating the integrated influence of evaporator architecture on coupled heat, liquid, salt, and vapor transport. However, because height and diameter were not varied independently, the present comparison does not isolate the individual effects of height, diameter, or Γ_*A*_. Instead, it evaluates the overall transport response of the investigated architectural classes. The cylindrical samples are therefore used to establish the transport‐matching framework, rather than to limit its application to this geometry. A controlled parametric study in which height and diameter are varied independently would be required to quantify their individual contributions and identify geometry‐specific optimal aspect ratios. The sample composition and architecture are summarized in Table 1. The detailed fabrication process and sample characterization are provided in Tables S1 and S2.

**TABLE 1:** Summary of the composition and architectural design of the samples.

Sample name	Γ_*A*_ approx.	Composition/architecture
LEB‐PVA	1.22	Neat PVA
LEB‐G0.50	1.39	PVA/0.50% GNP
HEB‐PVA	2.43	Neat PVA
HEB‐G0.25	2.09	PVA/0.25% GNP
HEB‐G0.50	2.06	PVA/0.50% GNP
HEB‐G5.00	2.16	PVA/5.00% GNP
LEB‐BL1	1.42	Bilayer
LEB‐BL2	1.54	Bilayer
HEB‐BL	2.17	Bilayer

### 2.2 Structure and Transport Characteristics of the Model Evaporators

**FIGURE 1:**
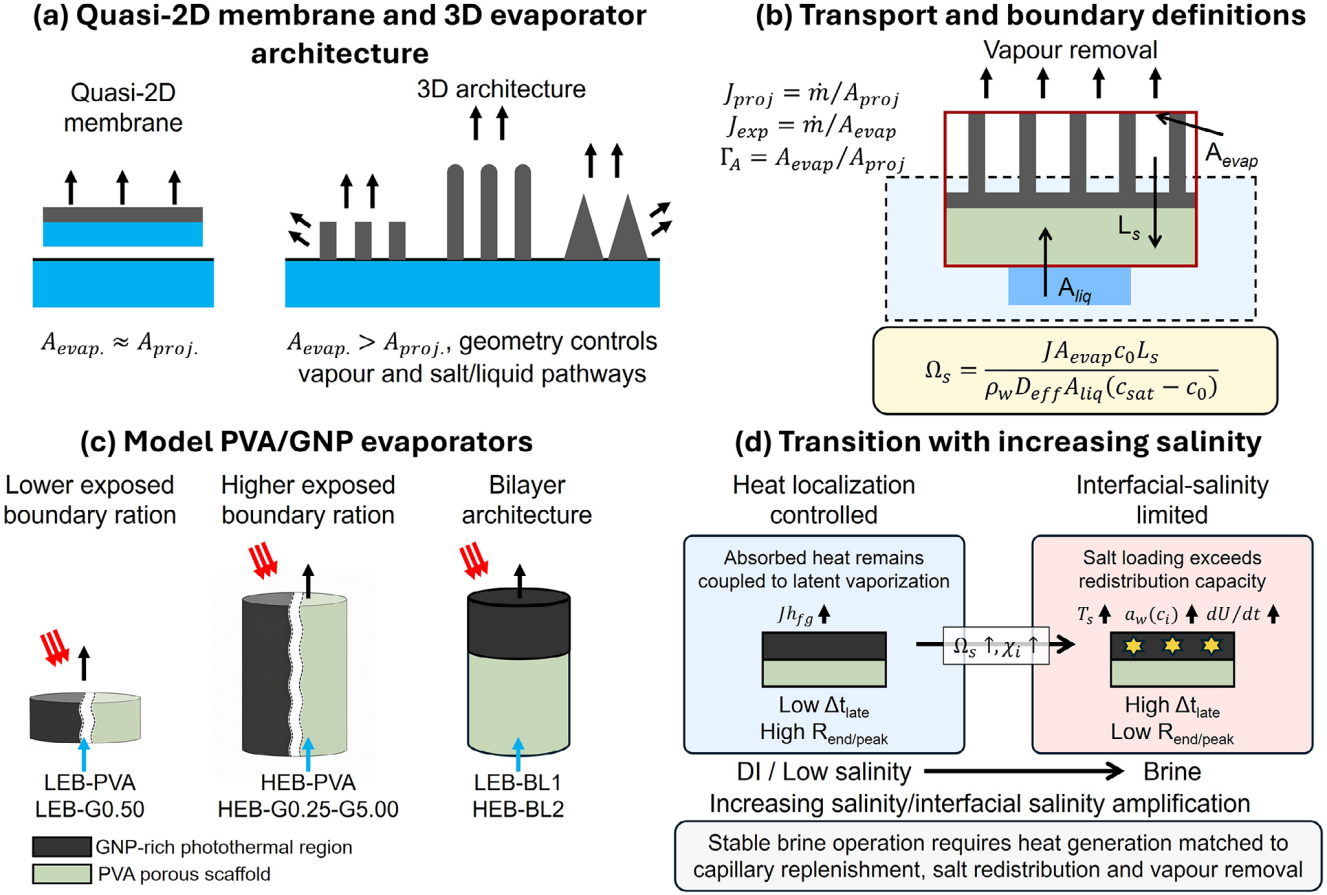
Interfacial salinity–transport matching framework for 3D evaporator architectures. (a) Conceptual comparison between a quasi‐2D membrane and representative 3D evaporator geometries, illustrating the emergence of non‐projected exposed boundaries and architecture‐dependent liquid/salt transport pathways. (b) General definitions of *A_proj_*, *A_evap_*, *A_liq_*, *L_s_*, Γ_*A*_, *J_proj_*, *J_exp_* and Ω_*s*_. For complex architectures, these descriptors are determined from the actual evaporation and liquid/salt transport domains. (c) Cylindrical PVA/GNP evaporators used as the model experimental system. (d) Transition from heat‐localization‐controlled operation at low salinity to interfacial‐salinity‐limited brine operation.

In quasi‐2D evaporators, evaporation is commonly reported with respect to the projected illuminated surface, and the lateral boundaries are usually small compared with the top surface. In 3D evaporators, however, finite height and exposed side surfaces introduce additional non‐projected boundaries that can participate in vapor removal, heat exchange, and liquid/salt transport. Figure 1a illustrates this architectural transition from a quasi‐2D membrane to a 3D evaporator with additional exposed boundaries.

As illustrated conceptually in Figure 1a, in this study, cylindrical evaporators were used as simplified model 3D architectures to examine these coupled boundary effects under a well‐defined geometry. Unlike quasi‐2D membranes, the cylindrical samples possess finite height, exposed lateral surfaces, and internal porous transport pathways, such that evaporation, heat exchange, and salt redistribution cannot be represented by the projected top surface alone. The cylindrical geometry provides a model system in which *A_proj_*, *A_evap_*, *A_liq_*, and *L_s_* can be determined without the additional geometric complexity associated with pillar arrays, conical structures, lattices, mushroom‐shaped units, or hierarchical porous sponges. This distinction motivates the use of the exposed‐boundary factor, Γ_*A*_ = *A*_evap_ /*A*_proj_, as an architectural descriptor for comparing 3D evaporator configurations.

The fabricated PVA/GNP evaporators formed highly porous interconnected architectures with vertically aligned channels that promoted continuous liquid transport between the bulk reservoir and evaporation interface (Figure S1). GNP incorporation reduced the average pore size without substantially changing the overall porosity, while excessive loading (G5.00) led to local agglomeration. The bilayer structures also showed continuous interfacial integration without visible delamination. UV–Vis–NIR analysis further confirmed that GNP incorporation significantly enhanced broadband solar absorption, consistent with the stronger dry photothermal response of the GNP‐containing evaporators (Figure S2a). Rapid water uptake was confirmed by time‐resolved droplet‐absorption images and transient contact‐angle measurements (Figure S2b). Water droplets were absorbed by the porous PVA‐based evaporators within less than 1.2 s, indicating fast capillary wetting and hydrophilic liquid‐transport pathways. Additional FTIR, Raman, XRD, and TGA analyses confirmed successful crosslinking, preservation of the graphitic structure of GNPs after thermal processing, and thermal stability within the operating range of the present SDIE system (Figure S3). Detailed structural and material characterization is provided in the Supplementary Information (Table S3).

### 2.3 Interfacial Salinity–Transport Matching Framework

Brine evaporation couples photothermal heat generation with interfacial salt enrichment, capillary replenishment, and vapor removal. At the evaporating interface, water is removed while salt remains in the liquid phase. The local interfacial salinity, *c_i_*, can therefore exceed the feed salinity, *c*_0_, before visible crystallization occurs.

The evaporation flux (*J*) is affected by surface temperature (*T_s_*), water activity at the evaporating interface (*a_w_* (*c_i_*)), saturation vapor density at the surface temperature (ρ_*v*,*sat*_(*T_s_*)), vapor mass‐transfer coefficient (*h_m_*), and ambient vapor density (ρ_*v*,∞_). Here, *h_m_* is determined experimentally from DI‐water evaporation under the same geometry and ambient conditions and then used to compare saline and brine operation (Table S4). The term *a_w_* (*c_i_*)ρ_*v*,*sat*_(*T_s_*) represents the salinity‐corrected vapor density at the interface. A rise in *T_s_* does not necessarily increase *J* when water activity at the evaporating interface decreases or when vapor removal and liquid replenishment become limiting.

Salt redistribution inside the porous architecture follows the advection–diffusion balance:

(1) $$\frac{\partial c}{\partial t}+u\cdot \nabla c=\nabla \cdot \left(D_{eff}\nabla c\right)$$

where *D_eff_* is the measured effective salt diffusivity in the porous network. Evaporation‐driven salt loading at the interface scales with:

(2) $$\overset{.}{\underset{salt,int}{m}}=c_{i}\frac{JA_{evap}}{\rho_{w}}$$

where *A_evap_* is the measured effective evaporative boundary and ρ_*w*_ is the water density. Salt back‐transport scales with:

(3) $$\overset{.}{\underset{salt,back}{m}}=D_{eff}A_{liq}\frac{\left(c_{i}c_{0}\right)}{L_{s}}$$

where *A_liq_* is the liquid/salt transport cross‐section and *L_s_* is the characteristic salt‐transport length. Similar balances have been used in 3D salt‐rejecting evaporators, where bridge number, bridge cross‐section, concentration difference, and transport length determine whether salt backflow maintains a low‐salinity evaporation surface [10].

Equations (2) and (3) describe the instantaneous scaling of evaporation‐driven salt loading and diffusive salt back‐transport using the evolving interfacial concentration, *c_i_*. To compare evaporator architectures on a common pre‐saturation basis, a reference salt‐loading/transport‐capacity number is defined using corresponding loading and redistribution scales. The nominal evaporation‐driven salt‐loading scale is evaluated using the feed salinity as 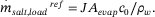


The maximum diffusive salt‐redistribution capacity before interfacial saturation is estimated using the largest available concentration difference (*c_sat_* − *c*_0_), via 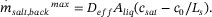 The salt‐loading/transport‐matching number is then defined as:

(4)

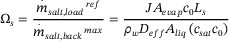




Here, *c*_sat_ is the saturation salinity at the relevant operating temperature. Ω_*s*_ is therefore a reference transport‐capacity number rather than an instantaneous interfacial flux ratio. A value approaching or exceeding unity indicates that the nominal salt loading is comparable to or greater than the maximum estimated diffusive redistribution capacity.

A value below unity indicates available redistribution capacity according to this scaling, but does not independently guarantee brine‐stable operation because capillary advection, transient concentration gradients, and other transport processes remain coupled. Low Ω_*s*_ indicates that salt redistribution capacity is sufficient relative to evaporation‐driven salt loading, whereas high Ω_*s*_ indicates that salt loading approaches or exceeds the available redistribution capacity (Figure 1b). This condition promotes interfacial salinity amplification, reduced water activity, weakened evaporative cooling, and late‐stage thermal drift (Figure 1c).

The salinity term *c*_0_/(*c_sat_* − *c*_0_) makes brine operation distinct from seawater‐level evaporation. As *c*_0_ approaches *c_sat_*, the salt load increases while the concentration margin for back‐diffusion decreases. A structure that remains evaporation‐cooled at 3.5 wt.% NaCl can therefore become transport‐limited at 20 wt.% NaCl without any change in photothermal composition. Setting Ω_*s*_ =  1 gives an architecture‐dependent critical salinity:

(5) $$c_{0}^{*}=\frac{c_{sat}}{1+\frac{JA_{evap}L_{s}}{\rho_{w}D_{eff}A_{liq}}}\;$$

High evaporation flux, large exposed evaporative boundary, long salt‐transport pathway, low effective diffusivity, and limited liquid/salt transport area shift the evaporator toward transport‐limited operation. Brine‐stable operation requires interfacial salinity control and sustained evaporative cooling, not only high photothermal temperature or absence of visible salt crystals (Figure 1d).

For the cylindrical model evaporators used here, Γ_*A*_ = *A_evap_*/*A_proj_*  and Γ_*A*_ = (*A_top_* + *A_side_*)/*A_top_*  =  1 + 4*L*/*d*, where *L* is the sample height, and *d* is the diameter. For fins, pillars, lattices, bridges, channels, and folded surfaces, Γ_*A*_, *A_evap_*, *A_liq_* and *L_s_* must be determined from the actual architecture. These quantities capture the coupled evaporation boundary, vapor‐removal pathway, and salt/liquid transport route. Recent 3D evaporator analysis showed that projected‐area flux can exceed the nominal one‐sun solar‐thermal limit when non‐projected boundaries exchange heat with the environment and participate in vapor release [19]. Projected flux is therefore insufficient as a stand‐alone metric for 3D brine evaporators, particularly when the same geometry also controls interfacial salinity and salt redistribution.

### 2.4 Dry Photothermal Response: Heat‐Generation Capacity

**FIGURE 2:**
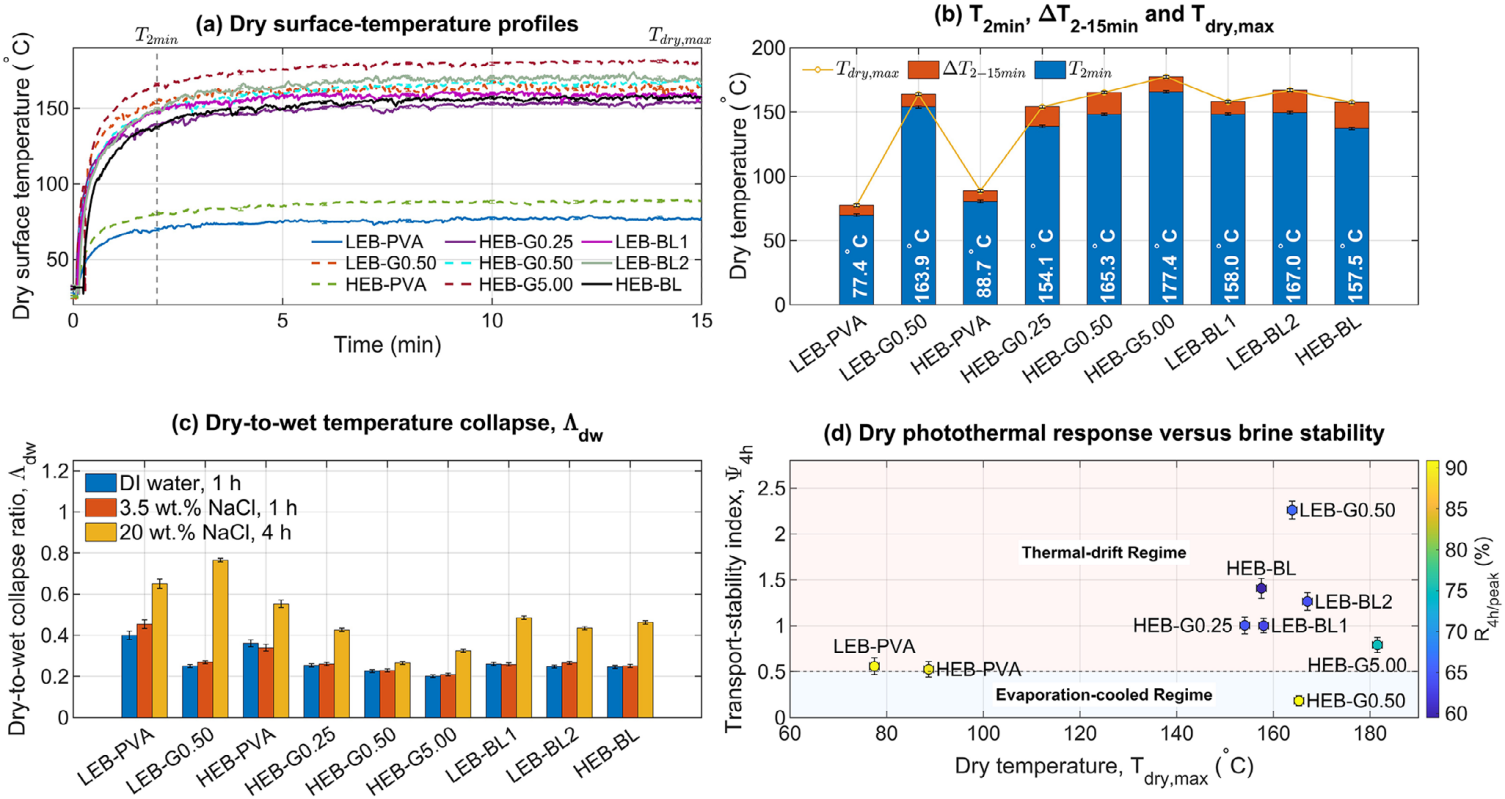
Dry photothermal response and dry‐to‐wet transition. (a) Dry surface‐temperature profiles. (b) *T*_2*min*_, Δ*T*_2 − 15*min*_and *T*_*dry*,*max*_. (c) Dry‐to‐wet temperature collapse, Λ_*dw*_, under DI water, 3.5 wt.% NaCl and 20 wt.% NaCl. (d) Dry photothermal response versus brine‐stability index, showing that the hottest dry sample is not necessarily the most brine‐stable sample. Data are presented as mean ± SD (*n* = 3 independent samples).

Figure 2 presents the photothermal response of the fabricated evaporators and examines whether dry‐state performance can predict subsequent brine stability. Figure 2a shows the dry photothermal response of the evaporators under solar irradiation. The dry test separates photothermal heat generation from water transport, latent heat consumption, and salt effects. All samples show a rapid temperature rise during the first ∼2 min, followed by a slower approach to a quasi‐steady temperature. The PVA‐only evaporators exhibit the weakest photothermal response, reaching final dry temperatures of 77.44°C for LEB‐PVA and 88.68°C for HEB‐PVA. GNP incorporation substantially increases the dry temperature. HEB‐G0.25, HEB‐G0.50, and HEB‐G5.00 reach 154.08, 165.28, and 177.36°C, respectively.

The reported dry temperatures correspond to local infrared surface temperatures of the evaporator rather than bulk‐liquid temperatures. Because no liquid feed was supplied during dry testing, latent heat removal was absent, and the high local temperatures of the GNP‐containing samples reflect strong broadband photothermal absorption, heat localization within the porous scaffold, and radiative, convective, and conductive heat losses. Thus, the dry test quantifies photothermal heat‐generation capacity rather than wet evaporation performance. The temperature response is governed by both architecture and photothermal loading: lower‐Γ_*A*_ samples heat rapidly during the initial stage, whereas higher‐Γ_*A*_ samples show stronger dry heat accumulation and higher final temperatures due to absorbed solar energy, thermal mass, and internal heat redistribution. Bilayer samples reach final dry temperatures of approximately 158°C to 170°C, indicating that a GNP‐rich upper region can localize heat near the irradiated surface even when supported by a neat PVA scaffold.

Figure 2b further separates the dry photothermal response into the initial photothermal activation (*T*_2*min*_) and the subsequent dry heat accumulation (Δ*T*_2 − 15*min*_). GNP‐containing samples show higher *T*_2*min*_ and higher final dry temperatures than PVA‐only evaporators. However, Δ*T*_2 − 15*min*_ should be interpreted as dry sensible heat storage after the initial photothermal activation, not as a measure of evaporation performance. This distinction is important because, under wet operation, the same absorbed heat can be removed by latent vaporization rather than stored as a temperature rise.

Under wet operation, absorbed heat should be consumed primarily as latent heat rather than stored as sensible heat:

(6) $$q_{abs}=Jh_{fg}+q_{loss}+\frac{dU}{dt}$$

where *Jh_fg_* is the latent heat used for evaporation and *dU*/*dt* is sensible heat storage. Efficient evaporation requires *Jh_fg_* to remain dominant. High dry temperature is beneficial only when the generated heat remains coupled to evaporation during wet operation. When *dU*/*dt* increases under brine conditions while flux retention decreases, the response corresponds to loss of evaporative cooling rather than improved evaporation.

The transition from dry photothermal heating to wet operation is quantified using the dry‐to‐wet temperature collapse ratio (Figure 2c):

(7) $$\Lambda_{dw}=\frac{T_{wet}-T_{\infty }}{T_{dry,max}-T_{\infty }}\;$$

where *T_wet_* is the wet operating temperature and *T*_∞_ is the ambient temperature. Lower Λ_*dw*_ indicates that the dry photothermal temperature is suppressed under wet operation, meaning that absorbed heat is effectively removed by latent vaporization. In contrast, higher Λ_*dw*_ during brine operation indicates that the wet surface temperature approaches the dry photothermal state, consistent with weaker evaporative cooling and greater sensible heat storage. This becomes more evident in Figure 2d, where the relationship between dry photothermal performance and subsequent brine operation is examined by correlating the maximum dry temperature with the transport‐stability index (Ψ). Samples with high dry photothermal temperature do not necessarily show low Ψ_4*h*_. For example, architectures with strong dry heating can still undergo late‐stage thermal drift and flux loss under brine.

Figure 2 establishes the distinction between dry heat‐generation capacity and brine stability. Although GNP incorporation increases the dry photothermal temperature, the dry‐to‐wet collapse ratio shows that absorbed heat can be removed by latent vaporization rather than stored as sensible heat during wet operation. Under brine conditions, samples with high dry temperatures can still undergo late‐stage thermal drift and flux loss. The results therefore demonstrate that dry heat‐generation capacity alone is not a reliable predictor of brine stability. Instead, stable brine operation requires the generated heat to remain coupled to liquid replenishment, salt redistribution, and latent heat removal. The dry photothermal response therefore defines heat‐generation capacity, but not the ability of the architecture to maintain coupled liquid replenishment, salt redistribution, and vapor removal.

### 2.5 Wet DI and Saline Operation: Evaporation‐Cooled Heat Localization

**FIGURE 3:**
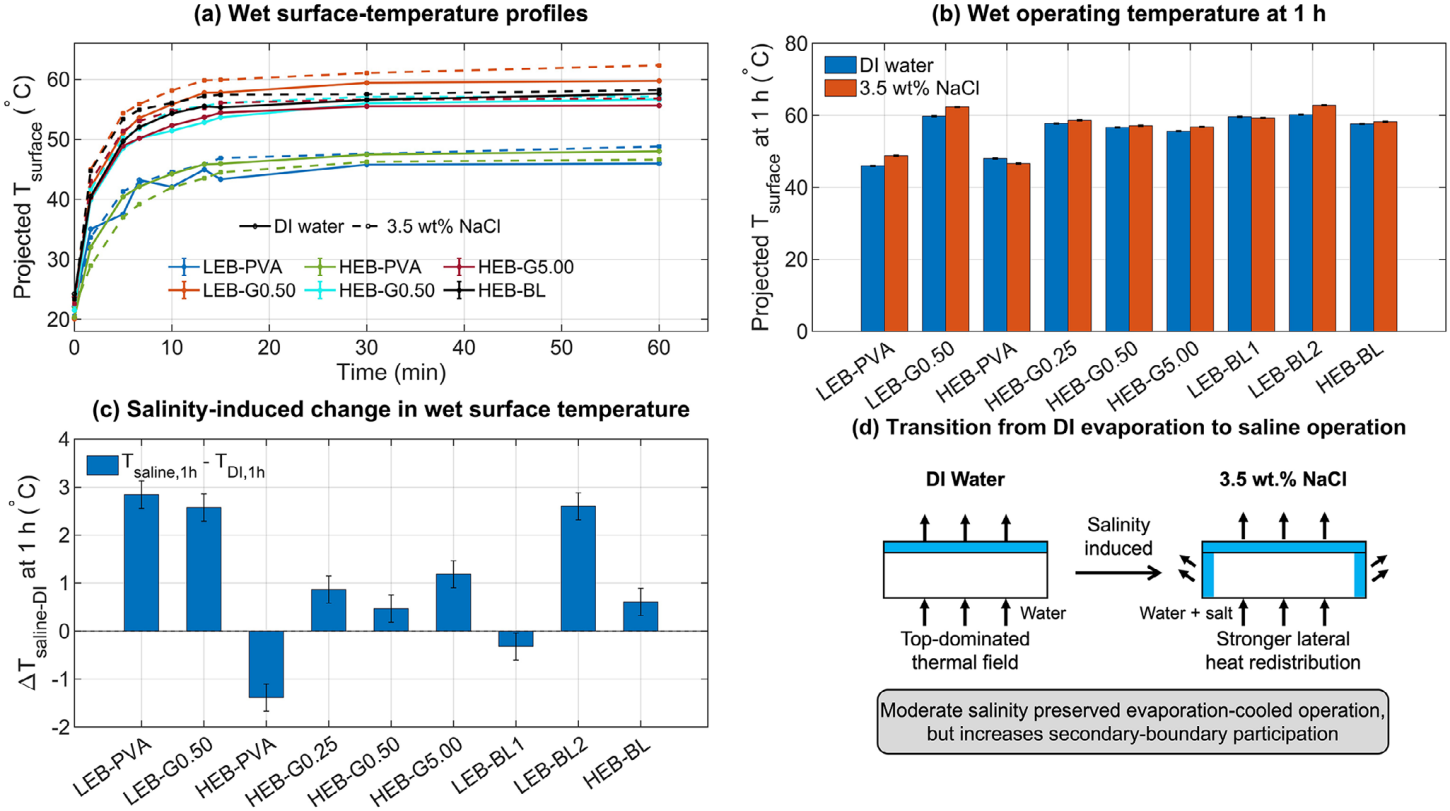
Wet‐state thermal response under DI water and saline water. (a) Wet surface‐temperature profiles under DI water and 3.5 wt.% NaCl. (b) Wet operating temperature at 1 h for DI water and 3.5 wt.% NaCl. (c) Salinity‐induced change in wet surface temperature, *T*_*saline*,1*h*_ − *T*_*DI*,1*h*_. (d) Transition from DI evaporation to saline operation. Data are presented as mean ± SD (*n* = 3 independent samples).

Wet operation changes the thermal response because absorbed solar energy is partly consumed by latent vaporization. As shown in Figure 3a, under DI‐water evaporation, GNP‐containing samples that reach 154–177°C in the dry state operate near 56–60°C after 1 h. HEB‐G0.25, HEB‐G0.50, HEB‐G5.00, and HEB‐BL reach 57.7°C, 56.6°C, 55.6°C, and 57.6°C, respectively. This demonstrates that evaporative cooling suppresses the dry photothermal contrast between samples.

Recent studies have reported lower surface temperatures for continuously wetted solar evaporators under one‐sun irradiation [20, 21]. For example, Shi et al. [20] reported an increase from 21°C to 31.7°C within 40 min for a wet melanin/cellulose‐based evaporator. In the present study, evaporative cooling similarly suppresses the dry photothermal response, with GNP‐containing samples operating at approximately 56°C to 60°C after 1 h under DI‐water evaporation despite reaching 154°C to 177°C when dry. Quantitative differences among studies may arise from variations in absorber composition, architecture, water‐supply configuration, support, ambient conditions, and infrared measurement procedures.

The DI‐water response represents evaporation‐cooled heat localization. Water supply remains sufficient to maintain latent heat removal, and the projected upper surface remains the dominant heated region. Under 3.5 wt.% NaCl, the final saline temperatures remain close to the DI‐water values for most samples (Figure 3b). The influence of moderate salinity on the wet thermal field is further quantified in Figure 3c through the salinity‐induced temperature change at 1 h. The temperature change remains modest compared with the later drift under 20 wt.% NaCl. For example, LEB‐PVA and LEB‐G0.50 show positive saline shifts of 2.8°C and 2.5°C, respectively, whereas HEB‐PVA shows a negative shift of −1.4°C. These small and architecture‐dependent changes indicate that moderate salinity perturbs the wet thermal field but does not create the severe transport limitation observed under brine operation, as schematically illustrated in Figure 3d.

Moderate salinity maintains the heat‐localization regime because interfacial salinity amplification remains limited and the concentration margin for salt redistribution remains large. Under brine, the term *c*_0_/(*c_sat_* − *c*_0_) increases and the same heat‐generation strategy can become transport‐limited. This behavior is consistent with confined‐water‐layer experiments where salinity in the evaporating layer increased rapidly before quasi‐steady operation, and where salinity evolution depended on evaporation, salt rejection, and natural convection [8].

Figure 3 identifies the evaporation‐cooled regime under DI water and moderate salinity. The GNP‐containing samples operate far below their dry photothermal temperatures, indicating that latent heat removal dominates the wet thermal response. The small temperature differences between DI water and 3.5 wt.% NaCl show that seawater‐level salinity perturbs the wet thermal field but does not produce the late‐stage transport limitation observed under 20 wt.% NaCl. The dominant transport constraint therefore shifts from heat localization under DI‐water and moderate‐salinity operation to coupled salt and liquid transport under brine conditions.

### 2.6 Brine Operation: Loss of Evaporative Cooling and Transport‐Limited Response

The 20 wt.% NaCl experiment was designed to probe the transition from evaporation‐cooled operation to transport‐limited brine operation. The 4 h endpoint was selected to compare Δ*T*_*late*,4*h*_, flux retention and transport‐stability behavior under identical brine stress. This time window should therefore be interpreted as a controlled mechanistic brine‐stress test rather than as evidence of long‐term field durability.

**FIGURE 4:**
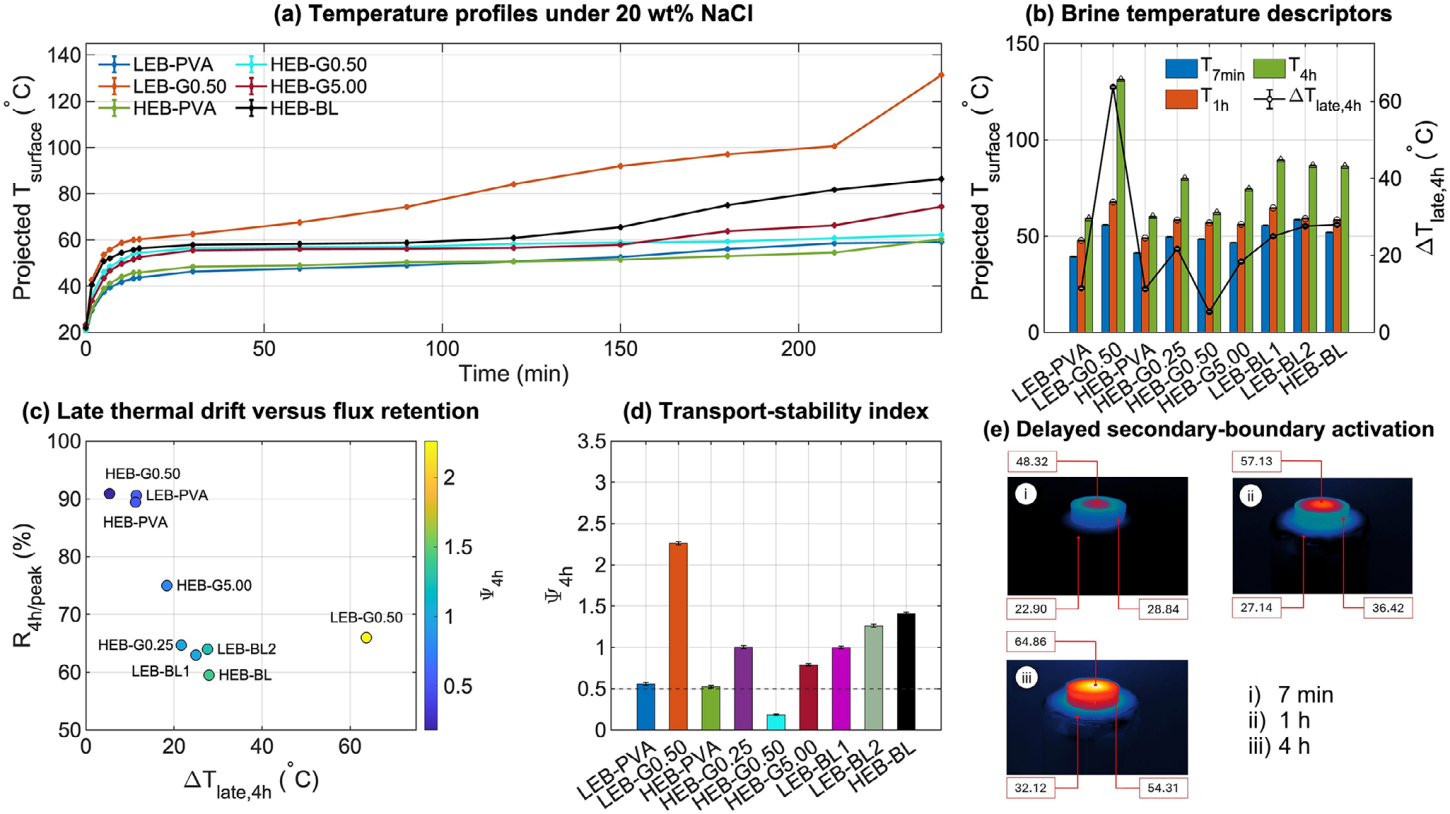
Brine‐induced loss of evaporative cooling. (a) Temperature profiles under 20 wt.% NaCl. (b) *T*_7*min*_, *T*_1*h*_, *T*_4*h*_and Δ*T*_*late*,4*h*_. (c) Relationship between Δ*T*_*late*,4*h*_and *R*_4*h*/*peak*_. (d) Transport‐stability index Ψ_4*h*_. (e) Representative thermal images showing delayed secondary‐boundary activation. Where error bars are shown, data are presented as mean ± SD (*n* = 3 independent samples).

Under this controlled brine‐stress condition, all samples heat rapidly during the first ∼7 min, reflecting early photothermal activation (Figure 4a). Thereafter, several architectures exhibited late‐stage thermal drift between 1 h and 4 h, indicating that the thermal response remained coupled to interfacial salt enrichment, capillary replenishment, and vapor removal. This delayed temperature rise provides an early indicator of transport limitation before surface crystallization becomes visually apparent. Time‐resolved infrared thermograms and optical photographs collected at hourly intervals are provided in Figure S5, confirming that the thermal drift precedes obvious salt accumulation and is accompanied by architecture‐dependent secondary‐boundary activation.

The 4 h endpoint captures a common brine‐stress state across the sample set before visible crystallization dominates the response. The thermal and flux data show architecture‐dependent differences during this period: Δ*T*_*late*,4*h*_ ranges from 5.34°C for HEB‐G0.50 to 63.68°C for LEB‐G0.50, while *R*_4*h*/*peak*_ ranges from 59.5% to 90.9% (Table S5). This endpoint therefore enables architecture‐to‐architecture comparison of the transition from evaporation‐cooled operation to transport‐limited brine response.

Figure 4b separates the brine thermal response into descriptors representing the initial photothermal activation (*T*_7*min*_), the evaporation‐cooled state (*T_ref_*), and the late transport‐limited response (*T_end_* and Δ*T*_*late*,*end*_). The post‐transient reference time is selected after the early evaporation‐cooled state has developed; in the present experiments, *t_ref_* =  1 h. The final test time is denoted as *t_end_*, which is 4 h in the present brine tests. The late‐stage brine thermal drift (Δ*T*_*late*,*end*_) is defined as the difference between *T_ref_* and *T_end_*, corresponding to *T*_1*h*_ and *T*_4*h*_ respectively.

The time‐normalized drift rate is:

(8) $$\beta_{T}=\frac{T_{end}-T_{ref}}{t_{end}-t_{ref}}\;$$

The use of *t_ref_*, *t_end_* and β_*T*_ allows comparison with brine tests performed over 6 h, 8 h, 24 h or multiple days. The same analysis can be applied to other endpoints by replacing *t_end_* and flux retention (*R*_*end*/*peak*_). Here, the flux retention is defined as:

(9) $$R_{end/peak}=\frac{J_{end}}{J_{peak}}\;$$

Which represents the ratio of the evaporation flux at the end of the test (*J_end_*), which in the present study corresponds to the fourth hour (*J*_4*h*_), to the maximum evaporation flux. Here, 4 h is used as the controlled endpoint for comparing architectures under equivalent high‐salinity stress.

Figure 4c directly compares late‐stage thermal drift with flux retention to determine whether heat accumulation is accompanied by evaporation decay. LEB‐G0.50 shows the strongest late‐stage drift. Its surface temperature increases from 67.6°C at 1 h to 131.3°C at 4 h, giving Δ *T*_*late*,4*h*_ =  63.7°C. This response coincides with reduced flux retention and is consistent with loss of evaporative cooling.

The local surface temperature of 131.3°C observed for LEB‐G0.50 after 4 h in 20 wt.% NaCl should not be interpreted as bulk‐liquid heating or stable high‐temperature evaporation. Instead, it indicates severe weakening of local evaporative cooling in the photothermal region. This response is consistent with brine‐induced transport limitation, interfacial salinity amplification, reduced water activity, insufficient liquid replenishment, flux decay, and increased sensible heat accumulation. The elevated temperature is therefore used as an indicator of heat‐to‐transport mismatch rather than as a performance advantage.

HEB‐G0.50 follows a different trajectory. Although its dry temperature is comparable to LEB‐G0.50, its brine temperature increases by only approximately 5°C between 1 h and 4 h, and it retains the highest brine‐flux stability. HEB‐G5.00 reaches the highest dry temperature, 177.4°C, but shows a larger brine drift of 18.4°C and lower flux retention. This comparison shows that increasing dry photothermal temperature does not necessarily improve brine stability.

The lower late‐stage thermal accumulation of HEB‐G0.50 is consistent with more effective matching among heat generation, interfacial water supply, salt redistribution, and vapor removal under the present test conditions. This interpretation is based on the integrated thermal, flux‐retention, salinity, and salt‐balance descriptors measured for the investigated sample set. Because the geometric and transport parameters were not varied independently, the contribution of any individual architectural feature cannot be isolated from the present results. The response of HEB‐G0.50 is therefore interpreted as an integrated transport behavior rather than evidence of a single causal design parameter.

HEB‐G0.50 retains ∼90.9% of its peak hourly brine flux by the fourth hour. In contrast, LEB‐G0.50 retains ∼66.0%, while several bilayer and GNP‐containing samples retain only ∼59–66%. The unstable samples therefore undergo simultaneous thermal drift and flux decay. This coupled response indicates that absorbed heat is increasingly stored or dissipated rather than converted into latent heat.

A transport‐stability index is defined as:

(10) $$\Psi_{end}=\frac{\beta_{T}}{R_{end/peak}}=\frac{\Delta T_{late,end}/\left(T_{ref}-T_{\infty }\right)}{J_{end}/J_{peak}}\;$$

Figure 4 shows that brine limitation develops after the early photothermal activation period. Figure 4d summarizes this response by combining late‐stage thermal drift and flux retention into the transport‐stability descriptor, Ψ. Low Ψ corresponds to evaporation‐cooled operation, whereas high Ψ indicates heat accumulation accompanied by flux loss. The divergence between samples during the 1–4 h interval therefore reflects differences in their ability to sustain latent heat removal as interfacial salinity increases. Samples with large late‐stage thermal drift and low flux retention remove a smaller fraction of absorbed heat through evaporation, leading to increased sensible heat accumulation and reduced brine stability. The contrast between LEB‐G0.50 and HEB‐G0.50 demonstrates that similar dry photothermal capacity can produce different brine responses when liquid supply, salt redistribution, and vapor removal are not equally matched. The representative thermal images in Figure 4e further show the delayed thermal response of the secondary exposed boundaries. Overall, Figure 4 establishes that brine‐stable operation is governed not by maximum dry temperature or maximum early flux alone, but by the ability of the evaporator architecture to maintain coupled heat, liquid, salt, and vapor transport under high‐salinity conditions.

### 2.7 Conductivity‐Constrained Interfacial Salinity Before Visible Salt Accumulation

The absence of visible salt crystals does not demonstrate that the evaporating interface remains at feed salinity. During brine evaporation, local salt concentration can increase near the liquid–vapor interface before crystallization appears. This enrichment reduces water activity and suppresses the effective vapor‐pressure driving force. Yang et al. [10] directly linked evaporation‐rate decline to increased evaporation‐surface salinity when mass‐transfer bridges were insufficient, and Zhang et al. [8] measured time‐dependent salinity increases in confined water layers during 3.5 wt.%, 10 wt.%, and 20 wt.% brine evaporation.

In the present work, local conductivity values were converted to local NaCl concentration using calibration solutions. The endpoint interfacial salinity was also checked against a salt mass balance based on the initial salt inventory, evaporated water mass, residual solution concentration, and any salt retained in/on the evaporator. This combined conductivity–mass‐balance approach provides experimental constraints on *c_i_* (*t_end_*) and distinguishes hidden interfacial salinity amplification from visible surface crystallization.

Taking *a_w_* = 1 for DI water provides the reference coefficient *h*_*m*,*DI*_ as follow:

(11) $$h_{m,DI}=\frac{J_{DI}}{\rho_{v,sat}\left(T_{DI}\right)-\rho_{v,\infty }}\;$$

**FIGURE 5:**
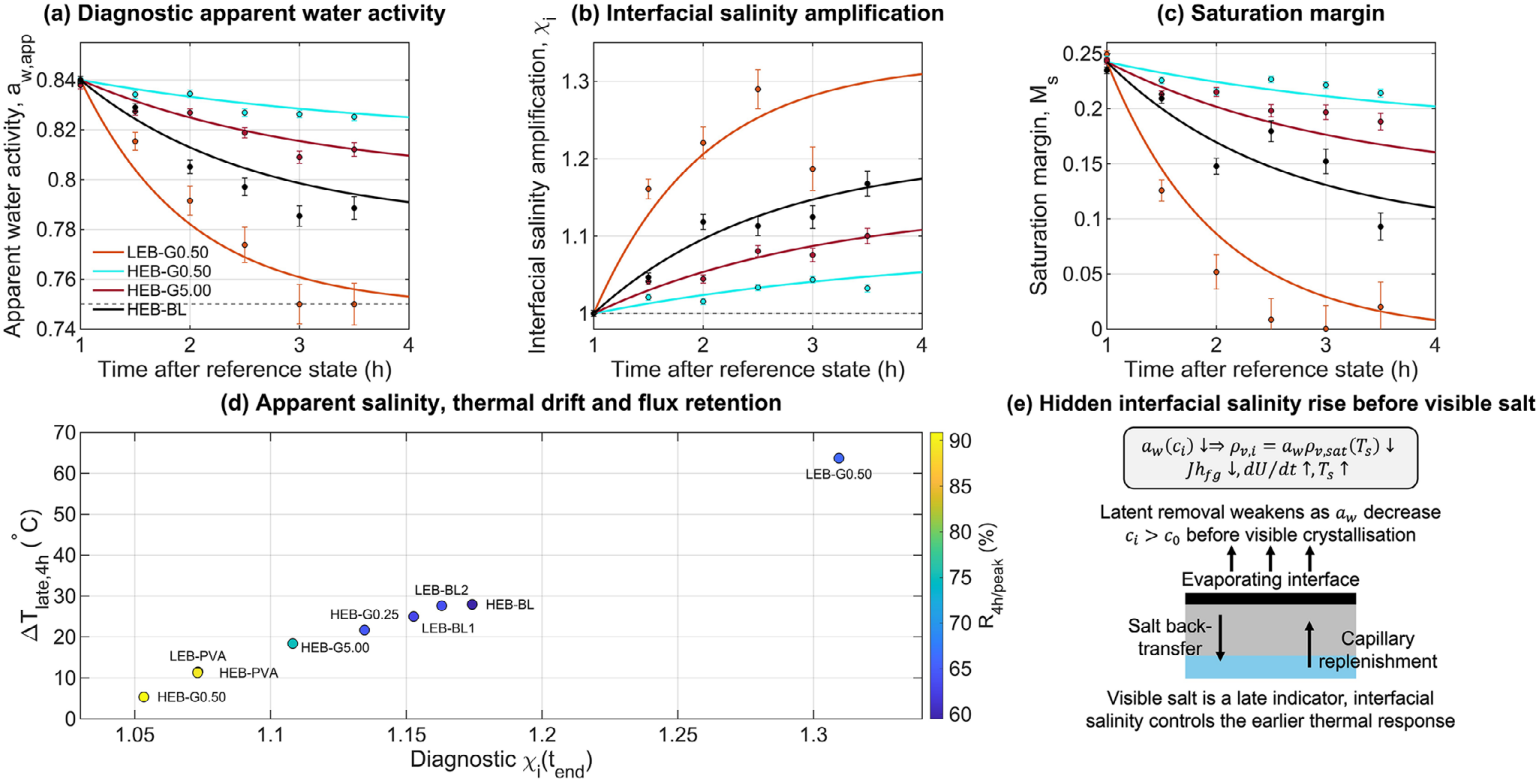
Conductivity‐constrained interfacial salinity analysis. (a) Apparent water activity, *a*_*w*,*app*_ (*t*), for selected stable and unstable samples. (b) Interfacial salinity amplification, χ_*i*_ (*t*), constrained by local conductivity and mass balance. (c) Saturation margin, *M_s_* (*t*). (d) Correlation between χ_*i*_ (*t_end_*), Δ*T*_*late*,*end*_ and *R*_*end*/*peak*_. (e) Hidden interfacial salinity rise reduces evaporative cooling before visible salt accumulation. Where error bars are shown, data are presented as mean ± SD (n = 3 independent samples).

Figure 5a evaluates the apparent water‐activity response using the measured brine temperatures and evaporation fluxes, while the endpoint interfacial salinity is independently constrained by local conductivity and salt mass balance.

For saline or brine operation, the salinity‐corrected apparent water activity is:

(12) $$a_{w,app}\left(t\right)=\frac{J\left(t\right)/h_{m,DI}+\rho_{v,\infty }}{\rho_{v,sat}\left(T_{s}\left(t\right)\right)}\;$$

The time‐dependent *a*_*w*,*app*_(*t*) contains gas‐side transport and liquid‐supply effects, but its endpoint is constrained by measured local conductivity and salt mass balance. It therefore serves as a conductivity‐constrained indicator of whether increasing surface temperature is accompanied by proportional vapor generation.

The interfacial salinity, shown in Figure 5b, is obtained from a NaCl water‐activity relation and local conductivity calibration. The interfacial salinity amplification factor is:

(13) $$\chi_{i}\left(t\right)=\frac{c_{i}\left(t\right)}{c_{0}}\;$$

The saturation margin, shown in Figure 5c, is defined as:

(14) $$M_{s}\left(t\right)=1-\frac{c_{i}\left(t\right)}{c_{sat}}$$

Figure 5d integrates the measured interfacial salinity amplification, salt mass balance, late‐stage thermal drift, and flux retention to evaluate the onset of transport‐limited brine operation. Stable brine operation is associated with low χ_*i*_, favorable salt balance, low Δ*T_late_* and high *R*_*end*/*peak*_. In contrast, high χ_*i*_, unfavorable salt balance, strong thermal drift, and low flux retention indicate a transition toward an interfacial‐salinity‐limited regime. These results show that a visually salt‐free surface is not sufficient to confirm brine stability. The evaporating interface must also maintain salt redistribution and liquid replenishment sufficient to sustain latent heat removal.

The mechanism is summarized schematically in Figure 5e. Conductivity‐constrained endpoint salinity and apparent water‐activity analysis indicate that the interface can enter a salt‐enriched state before visible crystallization becomes dominant. As local salinity increases, the saturation margin decreases, and the effective vapor‐pressure driving force is reduced. Evaporative cooling then weakens, leading to more pronounced late‐stage thermal drift. Figure 5 therefore supports the use of interfacial salinity amplification, salt balance, saturation margin, thermal drift, and flux retention as transport‐based descriptors of brine stability, rather than relying only on visual observation of salt crystallization.

### 2.8 Projected and Exposed‐Boundary Flux: Measured Side‐Boundary Contribution and Energy Consistency

The measured evaporation rate also depends on vapor removal from the evaporator to the ambient. For 2D/quasi‐2D evaporators, vapor removal can be approximated as top‐surface diffusion through an external boundary layer $\overset{.}{\underset{top}{m}}=D_{v}A_{proj}\Delta \rho_{v}/\delta$, where *D_v_* is the diffusivity of water vapor in air and δ is an effective diffusion‐layer thickness. A 3D evaporator introduces additional vapor‐removal pathways from non‐projected exposed boundaries:

(15) $$\overset{.}{\underset{3D}{m}}D_{v}A_{evap}\frac{\Delta \rho_{v}}{\delta_{eff}}$$

where *A_evap_* and δ_*eff*_ depend on architecture and ambient flow field. The effective influence of restricting the open lateral boundary was evaluated using side‐sealed controls. The unsealed configuration allowed vapor release from the top and side/exposed boundaries, whereas the side‐sealed configuration restricted non‐projected vapor release and left the projected top surface exposed while restricting the lateral boundary.

The lateral‐boundary effect was quantified using the fractional change in total evaporation rate between the unsealed and side‐sealed configurations:

(16) $$f_{side}=\frac{{\dot{m}}_{unsealed}-{\dot{m}}_{sealed}}{{\dot{m}}_{unsealed}}\;$$

where $\overset{.}{\underset{unsealed}{m}}$ and $\overset{.}{\underset{sealed}{m}}$ denote the total evaporation rates measured in the unsealed and side‐sealed configurations, respectively. The parameter *f_side_* therefore quantifies the fractional reduction in the measured total evaporation rate caused by restricting the lateral boundary. Larger values of *f_side_* indicate that the total evaporation rate is more sensitive to removal of the open side boundary.

The side‐sealed configuration does not isolate a single physical mechanism. In addition to suppressing lateral vapor removal, the sealing layer may modify sidewall radiative exchange, natural‐convective heat transfer, ambient heat uptake or loss, and transient heat storage. Consequently, *f_side_* is interpreted as an effective side‐boundary contribution under the applied sealing protocol, rather than as a direct measure of the sidewall vapor‐generation or vapor‐release fraction.

To estimate the possible scale of the associated thermal pathways, the sidewall heat‐exchange terms were evaluated by order‐of‐magnitude analysis. Because no forced airflow was applied, the natural‐convective term was estimated using *h_side_* =  2 W m^−2^ K^−1^. The infrared emissivity of the PVA/GNP evaporators was not independently measured; therefore, ε  =  0.95was adopted as an apparent emissivity for the order‐of‐magnitude radiative estimate.

For a sidewall warmer than the ambient and radiative surroundings, the natural‐convective and radiative heat‐loss rates are:

(17) $$Q_{conv,side}=h_{side}\;A_{side}\;\left(T_{side}-T_{\infty }\right)$$

(18) $$Q_{rad,side}=\varepsilon \;\sigma \;A_{side}\;\left(T_{side}^{4}-T_{sur}^{4}\right)$$

where *A_side_* is the lateral surface area, *T_side_* is the sidewall temperature, *T*_∞_is the ambient air temperature, *T_sur_* is the effective radiative‐surrounding temperature, εis the apparent emissivity, and σis the Stefan–Boltzmann constant. Under this sign convention, positive values represent heat loss from the sidewall, whereas negative values indicate environmental heat uptake when the sidewall is cooler than the surroundings. Exposed sidewalls therefore do not necessarily harvest environmental heat; the direction of heat exchange depends on the local sidewall‐to‐surroundings temperature difference.

Assuming ambient and radiative surroundings at 25°C and representative sidewall temperatures of 40–60°C, the combined natural‐convective and radiative heat loss is estimated to be approximately 0.12–0.31 kW m^−^^2^ per unit lateral area. For HEB‐G0.50, $\frac{A_{side}}{A_{proj}}=1.06$, corresponding to approximately 0.13–0.33 kW m^−^^2^ when expressed per projected area. This estimate is approximate because the spatial sidewall temperature distribution, natural‐convection coefficient, emissivity, and effective radiative‐surrounding temperature were not independently resolved. It is therefore not used to correct *f_side_*, determine a pure sidewall evaporation rate, or close the total energy balance.

The side‐sealed controls show that non‐projected boundaries measurably affect the total evaporation response. These observations are consistent with reported 3D open‐architecture evaporators, where partial enclosure reduced evaporation, demonstrating the importance of non‐projected vapor pathways and their associated thermal and mass‐transfer interactions [10].

**FIGURE 6:**
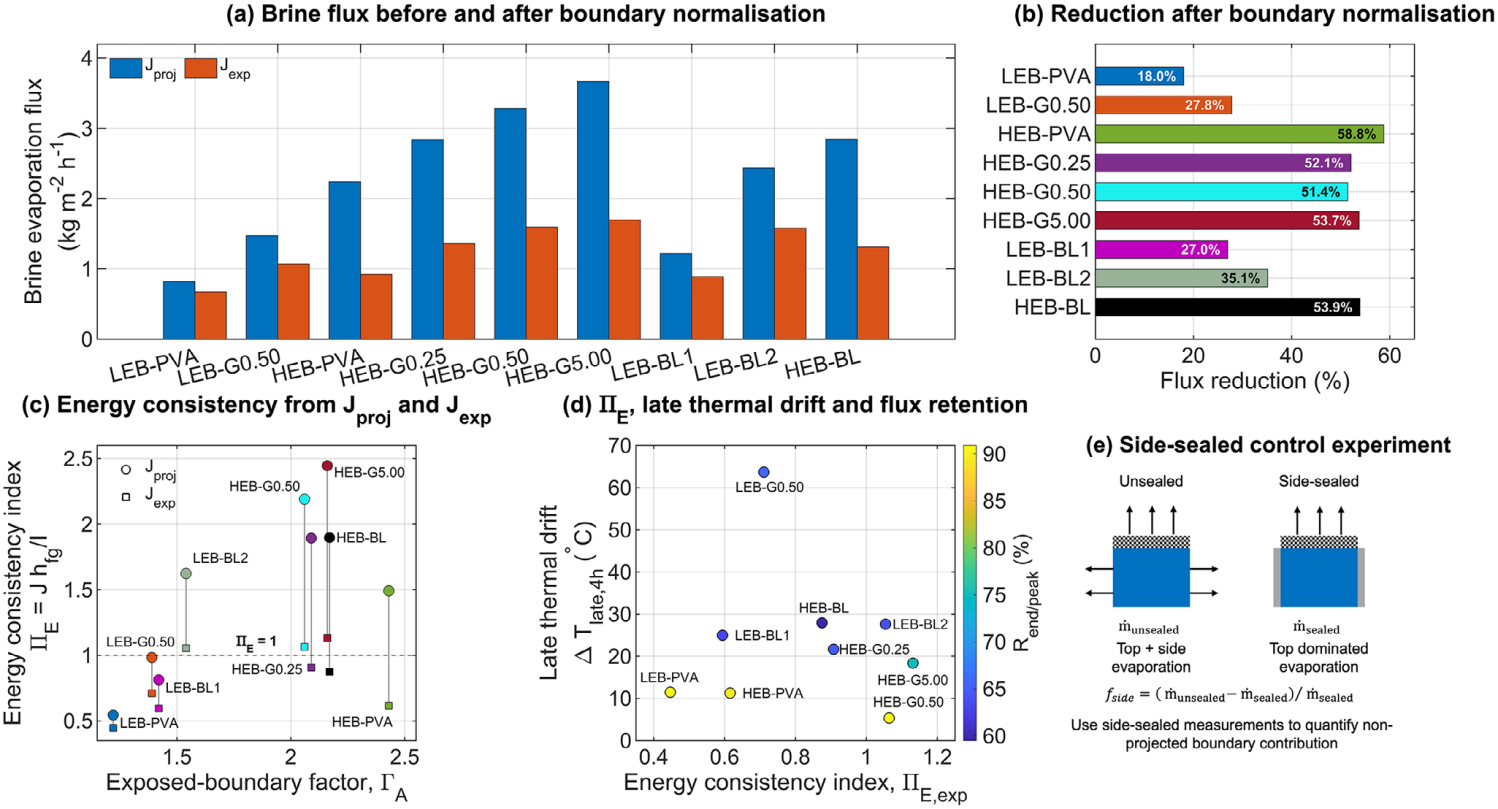
Energy consistency and boundary‐aware flux reporting. (a) Projected‐area and exposed‐boundary‐normalized brine flux. (b) Numerical change in the reported flux resulting from exposed‐boundary normalization. (c) Diagnostic latent‐demand indices calculated using projected‐area and exposed‐boundary‐normalized fluxes. (d) Relationship among the diagnostic energy index, thermal drift, and flux retention. (e) Side‐sealed and unsealed brine controls used to evaluate the effect of restricting the open lateral boundary on the total evaporation rate. The measured difference reflects suppression of lateral vapor release together with coupled changes in sidewall radiative exchange, natural convection, ambient heat uptake or loss, and transient heat storage.

Figure 6a compares the measured evaporation flux before and after exposed‐boundary normalization, while Figure 6b quantifies the corresponding reduction for each architecture. Figure 6c evaluates the energy consistency of the measured evaporation flux using the energy consistency index. Energy consistency is evaluated using the latent heat demand *q_lat_* =  *Jh_fg_*, and the corresponding energy number:

(19) $$\pi_{E}=\frac{Jh_{fg}}{I}\;$$

where *I* is the incident solar flux. A projected flux of approximately 3.2 kg m^−2^ h^−1^ corresponds to *q_lat_* 2.1 kW m^−2^, assuming *h_fg_* =  2.4 × 10^6^ J kg^−1^. Under one‐sun irradiation, this exceeds the projected solar input. The excess latent demand does not imply non‐thermal evaporation. It indicates that the projected area does not represent the full thermal boundary for a 3D evaporator. Non‐projected heat exchange, environmental heat uptake, transient storage, and exposed vapor‐release area contribute to the measured mass loss. Recent 3D evaporator analysis reached the same conclusion, showing that tall 3D evaporators can exceed the nominal solar‐thermal limit by absorbing environmental heat through cold sidewalls, with the extent depending on humidity and airflow [19].

Evaporation rates are therefore reported using both projected area and exposed evaporative boundary:

(20) $$J_{proj}=\frac{\dot{m}}{A_{proj}}\;$$

(21) $$J_{exp}=\frac{\dot{m}}{A_{evap}}\;$$

During full‐duration brine operation, converting from projected‐area flux to exposed‐boundary‐normalized flux reduces the calculated flux by 18.0% for LEB‐PVA and 27.8% for LEB‐G0.50 (Tables S6 and S7). In contrast, the reduction reaches 52.1% for HEB‐G0.25, 51.4% for HEB‐G0.50, 53.7% for HEB‐G5.00, and 53.9% for HEB‐BL. Boundary definition therefore significantly affects the apparent performance ranking of the 3D model evaporators.

The first‐hour brine data (Table S4) show the same effect. HEB‐G0.50, HEB‐G5.00, and HEB‐BL exhibit projected brine fluxes of approximately 3.17 kg m^−^^2^ h^−^^1^, 3.22 kg m^−^^2^ h^−^^1^, and 3.25 kg m^−^^2^ h^−^^1^, respectively. After exposed‐boundary normalization, these values decrease to approximately 1.54 kg m^−^^2^ h^−^^1^, 1.49 kg m^−^^2^ h^−^^1^, and 1.50 kg m^−^^2^ h^−^^1^. First‐hour projected flux therefore favors architectures with larger exposed boundaries during early photothermal operation. Figure 6d relates the exposed‐boundary energy‐consistency index to late‐stage thermal drift and flux retention, linking boundary‐aware flux normalization to transport stability. Figure 6e summarizes the unsealed and side‐sealed configurations used to evaluate the effective side‐boundary contribution.

Figure 6 clarifies how area definition affects reported 3D evaporation performance. Normalizing mass loss by the projected illuminated area gives higher apparent fluxes than normalization by the total exposed evaporative boundary, particularly for high‐exposed‐boundary architectures. This difference reflects the reporting basis, not a loss of intrinsic evaporation performance. The energy‐consistency analysis further shows that projected‐area normalization can imply latent heat demands exceeding the projected solar input, indicating that the effective thermal and vapor‐removal boundary extends beyond the projected top surface. Figure 6 therefore supports exposed‐boundary normalization as a boundary‐aware reporting approach for 3D evaporators while highlighting the limitations of relying only on projected‐area flux.

### 2.9 Reported Brine‐Stability Strategies Mapped onto the Transport‐Matching Criterion

Reported brine evaporators stabilize operation through different routes, including natural‐convection salt rejection, mass‐transfer bridges, localized crystallization, Janus separation, contactless evaporation, and multistage operation. These strategies differ in architecture, but they all regulate the same balance between heat generation, salt redistribution, liquid supply, and vapor removal. The key literature evidence supporting the interfacial salinity–transport matching framework, together with the corresponding observations from the present study, is summarized in Table 2.

**TABLE 2:** Literature evidence on the interfacial salinity–transport matching criterion.

Framework element	Literature evidence	Present result
Heat localization enables efficient evaporation but does not define brine stability	Heat‐localizing structures reached high solar thermal efficiency by combining absorption, insulation, hydrophilicity, and interconnected pores [22, 23].	GNP increases dry temperature to 150–180°C, but the hottest dry sample is not the most brine‐stable sample.
Salinity evolves before visible crystallization	Confined water‐layer salinity increased rapidly during 3.5 wt.%, 10 wt.%, and 20 wt.% brine evaporation before quasi‐steady behavior developed [8, 24].	Local conductivity and mass balance show interfacial salinity amplification before visible salt accumulation.
Surface salinity controls flux retention	Insufficient mass‐transfer bridges caused evaporation‐rate decline because evaporation‐surface salinity increased; sufficient bridges maintained stable evaporation over 12 h [10, 25].	LEB‐G0.50 shows high dry heating but only 66.0% flux retention; HEB‐G0.50 retains 90.9%.
Salinity threshold is architecture‐dependent	At high salinity, crystallization started when diffusion and convection backflow weakened [10, 26, 27].	The 20 wt.% NaCl test reveals transport mismatch not evident from DI or 3.5 wt.% NaCl response.
High‐salinity operation requires coupled heat and salt design	Confined‐water‐layer designs maintained high‐salinity operation by coupling thermal localization and salt rejection [8, 28].	HEB‐G0.50 shows low late‐stage drift and high retention, indicating stronger transport matching than LEB‐G0.50 or bilayers.
3D boundaries change flux interpretation	3D evaporators can exceed nominal solar‐thermal limits by absorbing environmental heat through non‐projected surfaces [19, 29].	HEB‐G0.50 gives high projected brine flux, but exposed‐boundary normalization reduces the calculated flux by 51.4%.
Open vapor pathways affect measured evaporation	Partial enclosure of 3D evaporators reduces evaporation by suppressing non‐projected vapor release [10, 30].	Side‐sealed controls directly quantify the non‐projected boundary contribution.
Visible salt is a late indicator	Salt‐localizing and ZLD strategies often manage where crystals appear, but interfacial salinity can rise before visible salt [31, 32, 33].	Conductivity‐constrained χ_*i*_ captures the pre‐crystallization transport state.

The reported strategies converge on one transport requirement: salt loading, capillary replenishment, and vapor removal must remain matched to heat generation. The present criterion expresses this requirement through χ_*i*_, Ω_*s*_, Δ*T_late_*, *R*_*end*/*peak*_, Ψ_*end*_, Γ_*A*_and $\pi_{E}$.

### 2.10 Brine Transport‐Matching Map and Design Rule

The measured thermal, flux, salinity, and boundary‐normalization data separate the evaporators into three operating regimes.

- In the dry photothermal regime, performance follows heat‐generation capacity. GNP loading dominates the temperature response, and higher dry temperature indicates stronger solar absorption.
- In the DI/saline evaporation regime, evaporative cooling moderates dry‐temperature differences. Heat localization remains important, but secondary‐boundary thermal participation begins to appear under saline water. The design remains primarily heat‐transfer controlled.
- In the brine transport‐limited regime, maximum heat generation can become counterproductive if evaporation‐driven salt loading exceeds salt diffusion and capillary replenishment. The relevant design objective shifts from maximum surface temperature to low interfacial salinity amplification, stable evaporative cooling, and high flux retention.

Based on these operating regimes, Table 3 compares the dry heat‐generation capacity, boundary‐normalization correction, late‐stage thermal drift, flux retention, and transport‐stability index for all evaporators under 20 wt.% NaCl. This comparison separates architectures that only generate high dry temperatures from those that maintain evaporation‐cooled, transport‐matched operation under brine conditions.

**TABLE 3:** Coupled dry photothermal, boundary‐normalized, and brine‐stability metrics under 20 wt.% NaCl.

Sample	*T*_dry, max _ (°C)	$\phi_{A}$ ^a^	Δ*T*_late, 4h _ (°C)	*R*_4h/peak _	Ψ_4h _	Brine‐response interpretation
LEB‐PVA	77.44	18.0	11.46	90.6	0.56	Low photothermal response; stable but low activity
LEB‐G0.50	163.88	27.8	63.68	66.0	2.26	Strong dry heating; severe loss of evaporative cooling
HEB‐PVA	88.68	58.8	11.26	89.4	0.53	Low photothermal response; large geometry correction
HEB‐G0.25	154.08	52.1	21.67	64.7	1.00	Strong heating; poor brine retention
HEB‐G0.50	165.28	51.4	5.34	90.9	0.18	Best transport‐matched response
HEB‐G5.00	177.36	53.7	18.38	75.0	0.79	Highest dry temperature; stronger brine drift
LEB‐BL1	157.98	27.0	24.97	63.0	1.00	Heat localization without stable brine retention
LEB‐BL2	167.04	35.1	27.62	64.0	1.26	High dry temperature; significant flux decay
HEB‐BL	157.52	53.9	27.95	59.5	1.41	Strong geometry correction and poor brine retention

^a^ $\phi_{A}=\left(1-J_{exp}/J_{proj}\right)\times 100\%$ is the percentage reduction in reported flux when the same mass‐loss rate is normalized by the exposed evaporative boundary rather than the projected illuminated area.

**FIGURE 7:**
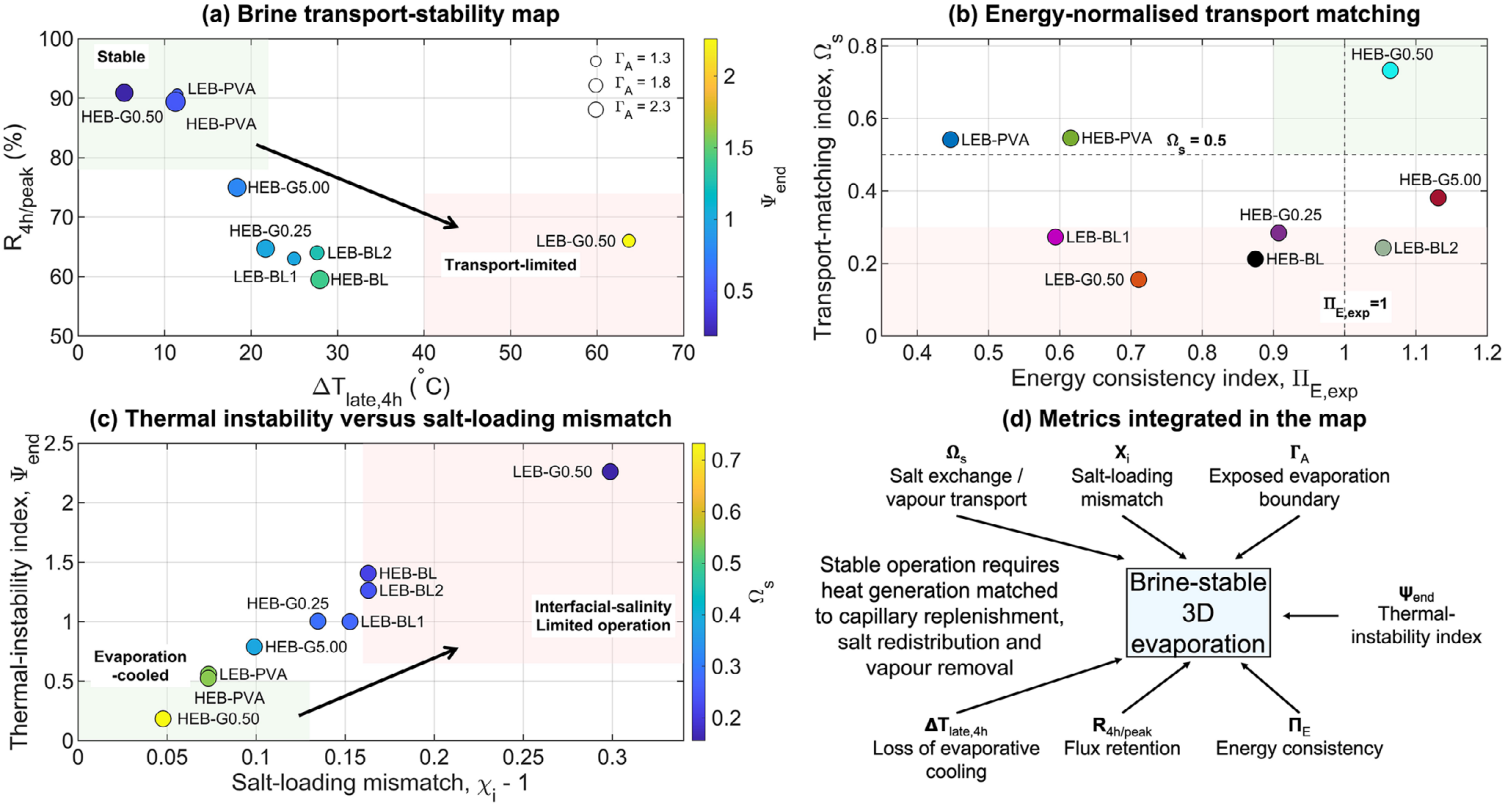
Brine transport‐matching map. (a) Brine transport‐stability map relating Δ*T*_*late*,4*h*_, *R*_4*h*/*peak*_, Ψ_*end*_and Γ_*A*_. (b) Energy‐normalized transport matching, relating $\pi_{E,exp}$ and Ω_*s*_. (c) Thermal instability versus salt‐loading mismatch, relating Ψ_*end*_, χ_*i*_ − 1and Ω_*s*_. (d) Metrics integrated in the map. The stable regime corresponds to low interfacial salinity amplification, low thermal drift, and high flux retention. The transport‐limited regime corresponds to high interfacial salinity amplification, high thermal drift, and low flux retention.

Figure 7 integrates interfacial salinity amplification, late‐stage thermal drift, flux retention, exposed‐boundary normalization, and energy consistency into a brine transport‐matching framework. Samples with low late‐stage thermal drift and high flux retention occupy the evaporation‐cooled regime, whereas samples with strong thermal drift and flux decay move toward the transport‐limited regime. The resulting map shows that neither dry photothermal temperature nor exposed‐boundary factor alone defines brine stability. Stable brine operation requires simultaneous matching of heat generation, capillary replenishment, salt redistribution, and vapor removal.

Figure 7a summarizes the brine response by combining late‐stage thermal drift, flux retention, transport stability, and exposed‐boundary factor. The comparison between LEB‐G0.50 and HEB‐G0.50 highlights the effect of architecture within the same GNP loading. Both samples contain 0.50 wt.% GNP and show strong dry photothermal responses, but LEB‐G0.50 undergoes severe late‐stage heating and flux decay, whereas HEB‐G0.50 remains thermally stable and retains flux. The comparison between HEB‐G0.50 and HEB‐G5.00 shows that increasing GNP loading within a similar exposed‐boundary class increases dry temperature but reduces brine stability. The bilayer samples further show that spatial heat localization does not guarantee brine‐flux retention unless salt and water transport remain matched to heat generation.

Within the investigated sample set, HEB‐G0.50 exhibited the most favorable measured transport‐matching response under 20 wt.% NaCl, combining the lowest late‐stage thermal drift of 5.34°C with the highest fourth‐hour flux retention of 90.9% among the GNP‐containing architectures. This classification is based on the combined thermal, flux‐retention, conductivity‐constrained salinity, salt‐balance, and transport descriptors measured under the present operating conditions.

The comparison between LEB‐G0.50 and HEB‐G0.50 shows that similar GNP loading and dry photothermal response can lead to substantially different brine behavior. This difference should not be attributed to a single isolated design parameter. Instead, it reflects the integrated effect of material composition, external geometry, exposed‐boundary participation, capillary replenishment, salt redistribution, vapor removal, and heat exchange. Dedicated future studies combining controlled experiments and computational modeling will be required to decouple the individual contributions of pore architecture, surface chemistry, height, diameter, effective thermal conductivity, and transport‐path geometry.

Increasing the exposed boundary or height‐to‐diameter ratio therefore does not necessarily improve evaporation performance. Larger sidewalls provide additional evaporation surfaces and vapor‐removal pathways, but they may also redistribute heat from the top photothermal region toward cooler lateral regions, introduce sidewall radiative and convective heat exchange, and extend liquid‐ and salt‐transport pathways. The effective wet thermal conductivity and top‐to‐sidewall heat‐transfer rate were not independently measured in this study. Therefore, an optimal aspect ratio cannot be extracted from the present dataset and is expected to be architecture‐, material‐, and condition‐dependent.

Figure 7b relates the energy‐consistency index to the salt‐loading/transport‐matching number, illustrating the balance between energy utilization and salt‐transport capability. Figure 7c correlates thermal instability with salt‐loading mismatch, showing the transition from evaporation‐cooled to interfacial‐salinity‐limited operation. Figure 7d schematically summarizes how the experimentally measured descriptors are integrated into the proposed framework.

Brine evaporator design should not be defined by maximum first‐hour evaporation rate or the absence of visible salt crystals alone. A brine‐stable evaporator must maintain low interfacial salinity amplification, low late‐stage thermal drift, high flux retention, and energy‐consistent evaporation over time. This requires controlled heat generation, sufficient liquid/salt transport area, short or effective salt‐redistribution pathways, high effective diffusivity/permeability, capillary replenishment, salt back‐diffusion or relocation routes, and vapor removal that does not over‐concentrate the evaporating interface.

Although demonstrated here using PVA/GNP evaporators, the proposed salinity–transport matching framework is not based on PVA or GNP chemistry. The salt‐loading/transport‐matching number, Ω_*s*_, is formulated from measurable evaporation and salt‐transport parameters, including evaporation flux, exposed evaporative area, feed salinity, effective salt diffusivity, liquid/salt transport area, transport length, and saturation concentration margin. The transport‐stability index, Ψ, is likewise based on normalized late‐stage thermal drift and flux retention during brine operation. These descriptors are material‐, architecture‐, and condition‐dependent in value, but their formulations can be applied to other porous solar evaporators when the corresponding thermal and transport parameters are determined under defined operating conditions.

The framework is also not restricted to cylindrical evaporators. The cylindrical samples used in this work provide a controlled model system for evaluating exposed‐boundary effects and salinity–transport matching, but the same descriptors can be defined for more complex 3D architectures, including pillar arrays, conical structures, hierarchical porous sponges, mushroom‐shaped units, bridges, fins, and folded surfaces. In these systems, *A_evap_*, *A_liq_*, *L_s_*, *D_eff_*, *c_i_*, late‐stage thermal drift and flux retention should be determined from the actual geometry and liquid‐connected transport pathways. For example, pillar or conical arrays may introduce multiple parallel evaporation surfaces and salt‐redistribution pathways, changing the local values and spatial distributions of *A*_evap_, *A*_liq_, *L_s_*,. These geometric changes alter the numerical transport balance, but not the underlying conservation‐based criterion that evaporation‐driven salt loading must remain matched by liquid replenishment and salt redistribution.

The present experiments were performed under controlled simulated solar irradiation to isolate architecture‐dependent transport behavior from fluctuations in irradiance, ambient temperature, humidity, and airflow. This controlled approach is appropriate for establishing and comparing the salinity–transport matching behavior because the key descriptors, including late‐stage thermal drift, flux retention, interfacial salinity amplification, exposed‐boundary‐normalized flux, and energy consistency, are directly affected by external boundary conditions. Under outdoor operation, the same descriptors can be evaluated over defined time windows if irradiance, ambient temperature, relative humidity, airflow, evaporation rate, surface temperature, and salinity response are recorded together. Outdoor testing is therefore required for device‐level validation and long‐term performance assessment, whereas controlled indoor brine‐stress testing provides a reproducible basis for developing and comparing the transport‐matching framework.

## 3 Conclusion

This study establishes an interfacial salinity–transport matching criterion for 3D solar evaporator architectures. The operating constraint changes from heat‐localization‐controlled evaporation under DI water and moderate salinity to salt/liquid‐transport‐limited evaporation under high‐salinity brine.

GNP incorporation increases dry photothermal temperature from approximately 70–90°C for neat PVA to 150–180°C for GNP‐containing samples. Under DI water and 3.5 wt.% NaCl, evaporative cooling reduces the temperature difference between samples and maintains comparatively stable operation. Under 20 wt.% NaCl brine, several samples show delayed heat accumulation after the early operating period. LEB‐G0.50 reaches 131.34°C after 4 h, whereas HEB‐G0.50 increases by only 5.34°C between 1 h and 4 h despite similar dry photothermal response.

Within the investigated sample set, HEB‐G0.50 exhibited the most favorable measured transport‐matching response under 20 wt.% NaCl, combining low late‐stage thermal drift with high flux retention. This classification is based on the integrated thermal, flux, salinity, salt‐balance, and transport descriptors measured under the present operating conditions. The result reflects the combined influence of material composition, geometry, and coupled heat–liquid–salt–vapor transport pathways, rather than an independently isolated contribution from any single design variable. Controlled experimental and computational studies in which these parameters are independently varied would be required to establish their individual contributions.

The divergence between dry photothermal ranking and brine‐stability ranking identifies heat‐to‐transport mismatch as the limiting process. Brine operation is controlled by evaporation‐driven salt loading, interfacial salinity amplification, water‐activity loss, capillary replenishment, vapor removal, and energy consistency. Local conductivity and salt mass balance confirm that the interface can become salt‐enriched before visible salt accumulation appears. Increasing salinity shifts the governing constraint from heat generation to coupled salt and water transport.

Evaporation performance also depends on the thermal and vapor boundary. For 3D architectures, exposed non‐projected boundaries contribute to heat exchange and vapor removal. Side‐sealed controls show that the non‐projected boundary contribution is experimentally measurable. Full‐duration brine flux decreases by approximately 51–54% for the high‐Γ_*A*_ GNP‐containing samples when calculated using exposed evaporative boundary rather than projected area. Energy consistency analysis further shows that high projected‐area flux can imply latent heat demand exceeding projected solar input, indicating an incomplete boundary definition rather than intrinsically superior evaporation.

The results support a design rule for 3D solar evaporator architectures: saline‐water evaporators can prioritize heat localization and vapor release, whereas brine evaporators require interfacial salinity control, capillary replenishment, salt redistribution, and sustained evaporative cooling. These results show that projected‐area flux and visible salt suppression should be reported together with local salinity, salt mass balance, late‐stage thermal drift, flux retention, exposed‐boundary normalization, and energy consistency.

## 4 Experimental Section

### 4.1 Materials

Polyvinyl alcohol (PVA; Mw = 89,000–98,000 g mol^−1^, ≥99% hydrolyzed; Aldrich) and graphene nanoplatelets (GNPs; Levidian, grade G3, non‐functionalized, carbon purity >99.9%) were used. G3 GNPs are produced by the LOOP technology of Levidian, which uses a microwave plasma process to convert methane into graphene and hydrogen, which itself enhances the sustainability effect of the study. More information about the G3 GNP is provided in Table S8 and Figure S6. Deionized water was used as the solvent. Sodium chloride was dissolved in deionized water to prepare 3.5 wt.% NaCl and 20 wt.% NaCl solutions.

### 4.2 Fabrication of PVA/GNP Evaporators

A 10% fraction of the required PVA was dissolved in deionized water at 60°C for 30 min. GNPs were added and dispersed by probe sonication for 30 min. The remaining PVA was added and dissolved under continuous stirring until a homogeneous precursor was obtained. The precursor was cast into molds with controlled dimensions to obtain low‐ and high‐exposed‐boundary samples. Bilayer samples were prepared by sequential casting of a GNP‐rich photothermal layer and a PVA‐rich support layer. The samples were frozen and freeze‐dried to produce porous evaporators. The final dimensions, dry mass, porosity, and exposed‐boundary factors were measured before testing. The fabrication parameters were determined based on literature review, optimization studies, and previous experimental experience [22, 34, 35, 36, 37].

### 4.3 Geometry, Exposed Evaporative Boundary and Liquid/Salt Transport Area

The projected area, *A_proj_*, was calculated from the illuminated top area. The exposed evaporative boundary, *A_evap_*, was obtained from the measured top and side/exposed surfaces that remained accessible to vapor release during wet operation. For cylindrical samples, *A_evap_* = *A_top_*  + *A_side_*, and Γ_*A*_ = *A_evap_* /*A_proj_*. The liquid/salt transport cross‐section, *A_liq_*, was determined from the water‐connected porous cross‐section available for capillary replenishment and salt redistribution. The characteristic salt‐transport length, *L_s_*, was determined from the measured distance between the evaporating region and the liquid reservoir/back‐diffusion pathway. The same definitions were used in the calculation of Ω_*s*_.

### 4.4 Effective Salt Diffusivity Measurement

Effective salt diffusivity, *D_eff_*, was measured for each sample using salt‐transport/ conductivity experiments. Water‐saturated samples were placed between solutions with a known concentration difference, and the time‐dependent conductivity change was recorded under controlled temperature. The measured conductivity response was converted to NaCl concentration using calibration solutions *D_eff_* was obtained by fitting the transient concentration evolution to the 1D diffusion model with the measured sample length and boundary concentrations. The extracted *D_eff_* values were used directly in Equation (5).

### 4.5 Solar Evaporation Tests

DI‐water, 3.5 wt.% NaCl and 20 wt.% NaCl evaporation tests were performed under simulated solar irradiation of 1 sun (1 kW m^−2^). Surface temperature was recorded using an infrared camera, and mass loss was recorded using an analytical balance. Ambient temperature, relative humidity, and airflow conditions were monitored during all tests. Dry tests were performed without liquid feed to quantify photothermal heat‐generation capacity. Wet tests were performed using DI water and 3.5 wt.% NaCl to establish evaporation‐cooled operation under low and moderate salinity. Brine tests were performed using 20 wt.% NaCl for 4 h as a controlled brine‐transport stress test. The duration was selected to separate the early photothermal activation regime from the later transport‐limited brine response. The initial few minutes primarily reflect heat generation and establishment of the evaporation‐cooled state, whereas the 1–4 h period allows architecture‐dependent differences in salt redistribution, capillary replenishment, late‐stage thermal drift, and flux retention to emerge. The 4 h endpoint therefore provides a reproducible comparison point for evaluating transport matching across the sample set. The test is not intended as a long‐term durability assessment, because longer operation may introduce additional effects such as extensive salt crystallization, pore blockage, optical shielding, and structural ageing. All tests were repeated at least three times using three independently prepared samples to confirm reproducibility. Further details are provided in Figure S4.

### 4.6 Local Conductivity and Salt Mass Balance

Local conductivity measurements were performed before and after brine operation for all samples. After the test, liquid was extracted from the evaporating/interfacial region and from the bulk reservoir where applicable. Conductivity was measured using a calibrated conductivity probe and converted to NaCl concentration using calibration standards prepared between 0 wt.% and 25 wt.% NaCl. Salt mass balance was calculated from the initial salt mass, evaporated water mass, residual bulk concentration, interfacial/local concentration, and any salt retained in or on the evaporator. The conductivity‐derived local salinity and mass balance were used to determine *c_i_* (*t_end_*), χ_*i*_(*t_end_*)and the saturation margin.

### 4.7 Side‐Sealed Control Experiments

Side‐sealed control experiments were performed to evaluate the influence of restricting the open lateral boundary on the total evaporation performance. In the unsealed configuration, both the projected top surface and the lateral boundaries remained open to vapor removal and heat exchange with the surroundings. In the side‐sealed configuration, the lateral surfaces were covered using a 100 µm‐thick transparent PVC binding‐cover film, while the projected top evaporation surface remained fully exposed. Therefore, the side‐sealed control is interpreted as an integrated lateral‐boundary test rather than an isolated measurement of sidewall vapor release. In addition to suppressing lateral vapor removal, the sealing layer may alter sidewall radiative exchange, natural‐convective heat transfer, ambient heat uptake or loss, and transient heat storage. Therefore, the measured parameter represents the effective contribution of the lateral boundary under the applied sealing method.

The evaporation rates $\overset{.}{\underset{unsealed}{m}}$ and $\overset{.}{\underset{sealed}{m}}$ were measured under identical illumination and ambient conditions. The effective side‐boundary contribution under the applied sealing method was calculated as $f_{side}=\left({\dot{m}}_{unsealed}-{\dot{m}}_{sealed}\right)/\overset{.}{\underset{unsealed}{m}}$.

### 4.8 Data Analysis

Projected‐area flux and exposed‐boundary‐normalized flux were calculated as $J_{proj}=\dot{m}/A_{proj}$ and $J_{exp}=\dot{m}/A_{evap}$, respectively. Energy consistency was evaluated using $\pi_{E}=Jh_{fg}/I$. The late‐stage thermal drift was calculated as Δ *T*_*late*,*end*_ = *T_end_*  − *T_ref_*, with *t_ref_* =  1 h and *t_end_* =  4 h for the present brine experiments. Flux retention was calculated as *R*_*end*/*peak*_ = *J_end_* /*J_peak_*. The transport‐stability index was calculated as Ψ_*end*_ =  [Δ*T*_*late*,*end*_/(*T_ref_* − *T*_∞_)]/*R*_*end*/*peak*_. The salt‐loading balance, Ω_*s*_, was calculated using measured *J*, *A_evap_*, *D_eff_*, *A_liq_*, *L_s_*, *c*_0_, *c_sat_* and conductivity‐constrained *c_i_*.

### 4.9 Statistical Analysis

All experiments were performed using three independently prepared samples, and each measurement was repeated three times to assess reproducibility. Reported values represent the mean of the independent measurements unless otherwise stated. Error bars shown in the figures represent ±SD calculated from independent measurements. No data transformation, normalization, or outlier exclusion procedures were applied prior to analysis.

For pore‐size characterization, the diameters of 50 individual pores were measured from SEM micrographs for each sample using ImageJ software (National Institutes of Health, USA). Porosity was determined by image analysis using the thresholding and area‐fraction functions in ImageJ. Differences in pore diameter between samples were evaluated using one‐way analysis of variance (ANOVA), with statistical significance accepted at *p* < 0.05. Statistical analyses were performed using IBM SPSS Statistics (Version 25).

The sample size (*n*) for each experiment is indicated in the corresponding figure captions, tables, or Supporting Information and was *n* = 3 unless otherwise stated.

## Nomenclature

**Symbols**
*A*
: Surface area, m^2^
*A_liq_*
: Effective liquid/salt transport cross‐sectional area, m^2^
*a_w_*
: Water activity, –
*c*
: Local salt concentration, Kg m^−3^
$c_{0}^{*}$
: Architecture‐dependent critical feed salinity, Kg m^−3^
*d*
: Sample diameter, m
*D_eff_*
: Effective salt diffusivity in the porous evaporator, m^2^ s^−1^
*D_v_*
: Water‐vapor diffusivity in air, m^2^ s^−1^
*h_fg_*
: Latent heat of vaporization, J kg^−1^
*h_m_*
: Vapor mass‐transfer coefficient, m s^−1^
*h_side_*
: Sidewall natural‐convection heat transfer coefficient, W m^−2^ K^−1^
*I*
: Incident solar irradiance, W m^−2^
*J*
: Evaporation flux, kg m^−2^ h^−1^ kg m^−2^ s^−1^
*L*
: Sample height (thickness), m
*L_s_*
: Characteristic salt‐transport length, m
*M_s_*
: Saturation margin, defined as 1 − *c_i_*/*c_sat_*, –
$\dot{m}$
: Total evaporation mass rate, kg s^−1^
$\overset{.}{\underset{salt,int}{m}}$
: Evaporation‐driven salt loading rate at the interface, kg s^−1^
*Q*_*conv*,*side*_
: Sidewall convective heat transfer rate, W
*Q*_*rad*,*side*_
: Sidewall radiative heat transfer rate, W
*q_abs_*
: Absorbed solar heat flux, W m^−2^
*R*
: Flux retention ratio, –
*t*
: Time, s, min or h
*T*
: Temperature, °C, K
*u*
: Liquid velocity inside the porous evaporator, m s^−1^
*dU*/*dt*
: Sensible heat storage rate, W m^−2^ or W

**Greek**
β_*T*_
: Time‐normalized late thermal drift rate, °C h^−1^
Γ_*A*_
: Exposed‐boundary factor, defined as *A_evap_*/*A_proj_*, –
δ: Vapor diffusion‐layer thickness for quasi‐2D evaporation, m
δ_*eff*_
: Effective vapor diffusion‐layer thickness for 3D evaporation, m
Δρ_*v*_
: Vapor‐density difference between interface and ambient, kg m^−3^
χ_*i*_
: Interfacial salinity amplification factor, defined as *c_i_*/*c*_0_, –
*K_local_*
: Local conductivity used to determine local salinity, mS cm^−1^
Λ_*dw*_
: Dry‐to‐wet temperature collapse ratio, –
Ω_*s*_
: Salt‐loading/transport‐matching number, –
$\pi_{E}$
: Energy consistency index, defined as *Jh_fg_*/*I*, –
ρ: Density, kg m^−3^
Ψ: Transport‐stability index, –
ε: Apparent infrared emissivity, –
σ: Stefan–Boltzmann constant (5.670×10^−8^), W m^−2^ K^−4^
$\phi_{A}$
: The percentage reduction in the evaporation flux, %

**Subscripts**
BL: Bilayer, –
DI: Deionized water, –
GNP: Graphene nanoplatelet, –
HEB: High exposed‐boundary class, –
IR: Infrared, –
LEB: Low exposed‐boundary class, –
Lat: Latent heat, –
NaCl: Sodium chloride, –
PVA: Polyvinyl alcohol, –
SDIE: Solar‐driven interfacial evaporation, –
v: Vapor, –
w: Water, –
wt.%: Weight percent, –

## Author Contributions

**Omid Doustdar**: investigation, validation, methodology, writing – review and editing. **Reza Nekouie Esfahani**: investigation, methodology, writing – review and editing. **Mohammad Sajad Sorayani Bafqi**: investigation, methodology, writing – original draft, data curation, formal analysis, writing – review and editing. **Ali Sadaghiani**: conceptualization, investigation, supervision, funding acquisition, project administration, writing – original draft, writing – review and editing. **Arun Prakash Aranga Raju**: investigation, writing – review and editing, methodology, validation, resources.

## Conflicts of Interest

The authors declare no conflicts of interest.

## Supporting information




**Supporting File**: advs77527‐sup‐0001‐SuppMat.docx.

## Data Availability

The data that support the findings of this study are available from the corresponding author upon reasonable request.
