# Supplementary material for: Interfacial Salinity–Transport Matching in 3D Solar Evaporators: A Framework for Brine‐Stable Solar Interfacial Evaporation
Source: Adv Sci (Weinh). 2026 Aug 30;13(60):e77527. doi: 10.1002/advs.77527 (PMC13526696; doi:10.1002/advs.77527)
Supplement: Supplementary file 1 [file ADVS-13-e77527-s001.docx]

**Supporting Information**

**Interfacial Salinity–Transport Matching in 3D Solar Evaporators: A Framework for Brine-Stable Solar Interfacial Evaporation**

**Mohammad Sajad Sorayani Bafqi^‡^, Arun Prakash Aranga Raju^§^, Omid Doustdar^‡^, Reza Nekouie Esfahani^||^, Ali Sadaghiani^‡,^***

**^‡^** SMaRT Research Group, Department of Mechanical Engineering, School of Engineering, University of Birmingham, Birmingham B15 2TT, United Kingdom.

^§^ Levidian Nanosystems Limited, Levidian Technology Centre, 17 Mercers Row, Cambridge, CB5 8HY, United Kingdom.

^||^The Manufacturing Technology Centre Ltd, Coventry CV7 9JU, United Kingdom.

* Corresponding author: [a.sadaghiani@bham.ac.uk](mailto:a.sadaghiani@bham.ac.uk)

# S1. Sample preparation

The samples were fabricated through a multi-step procedure. Initially, 10% of the required polyvinyl alcohol (PVA) was dissolved in deionized water (DI) at 60 °C for 30 min under continuous stirring using a hotplate stirrer (OHAUS Guardian 3000 Hotplate Stirrer). This pre-dissolved PVA fraction acted as a stabilizing medium, improving the dispersion of graphene nanoplatelets (GNPs) and reducing their aggregation and sedimentation during subsequent processing.

The required amount of GNPs was then added to the preliminary PVA solution and dispersed using a probe sonicator (Benchmark Pulse 150 Ultrasonic Homogenizer) for 30 min to obtain a homogeneous suspension. The remaining PVA was gradually added to the dispersion to achieve a final polymer concentration of 5 wt.%. The resulting mixture was stirred at 60°C for 2 h to ensure complete polymer dissolution and uniform nanofiller distribution, followed by cooling at room temperature for 1 h (while keeping the stirring) to stabilize the prepared solution. The compositions of the fabricated dispersions are summarized in **Table S1**.

**Table S1. Composition of PVA/GNP nanocomposite precursor solutions.**

| **Sample group** | **Description** | **PVA / wt.%** | **GNP loading / wt.%** |
| --- | --- | --- | --- |
| Neat PVA | Pure PVA solution | 5 | 0 |
| PVA/GNP | PVA/0.25% GNP | 5 | 0.25 |
| PVA/GNP | PVA/0.50% GNP | 5 | 0.50 |
| PVA/GNP | PVA/5.00% GNP | 5 | 5.00 |

For sample fabrication, 15 mL of the prepared dispersion was cast into beakers with different diameters, allowing the sample geometry and exposed-boundary factor to be varied while maintaining constant solution volume and composition. The filled beakers were subjected to freeze-casting by placing them overnight in the freezing chamber of a freeze dryer at −110°C (Scanvac CoolSafe Touch 110-4). Sublimation drying was then conducted under vacuum at 4°C for 48 h, forming porous hydrogel-derived aerogels.

For bilayer structures, a second layer of PVA/GNP dispersion was gently cast onto the frozen first layer after approximately 2 h of initial freezing to promote interfacial integration between layers. The total cast volume of the bilayer system was maintained at 15 mL. After freeze-drying, the aerogel samples were exposed to ambient conditions at room temperature and 40% relative humidity for 1 h to enable controlled moisture uptake before thermal crosslinking. Crosslinking was carried out in an oven (Genlab Prime Oven, MINO/30/TDIG/TIM/10PRO) by heating the samples to 140°C over 30 min, followed by a gradual temperature increase to 180 °C for 20 h. The samples were naturally cooled to room temperature and stored in a desiccator before characterization and evaporation testing. The fabrication parameters were selected based on literature, optimization studies and previous experimental experience [1–4].

# S2. Sample designation, composition and geometry

The sample names used in the main manuscript describe the exposed-boundary class and composition. LEB denotes the low exposed-boundary class, HEB denotes the high exposed-boundary class, G0.25, G0.50 and G5.00 denote GNP loading, and BL denotes bilayer architecture. The measured sample dimensions are summarized in **Table S2**.

**Table S2. Designation, composition and geometrical characteristics of fabricated evaporators.**

| **Sample name** | **Structure** | **Composition** | **Thickness / mm** | **Diameter / mm** |
| --- | --- | --- | --- | --- |
| LEB-PVA | Thin single layer | Pure PVA | 2.84 | 51.72 |
| LEB-G0.50 | Thin single layer | PVA/0.50% GNP | 4.57 | 47.43 |
| HEB-PVA | Thick single layer | Pure PVA | 11.26 | 31.60 |
| HEB-G0.25 | Thick single layer | PVA/0.25% GNP | 9.31 | 34.24 |
| HEB-G0.50 | Thick single layer | PVA/0.50% GNP | 8.98 | 33.91 |
| HEB-G5.00 | Thick single layer | PVA/5.00% GNP | 10.02 | 34.48 |
| LEB-BL1 | Thin bilayer | Top: PVA/0.50% GNP; bottom: pure PVA | 1.11 + 3.68 = 4.79 | 45.52 |
| LEB-BL2 | Thin bilayer | Top: PVA/0.50% GNP; bottom: pure PVA | 1.78 + 3.26 = 5.04 | 37.24 |
| HEB-BL | Thick bilayer | Top: PVA/0.50% GNP; bottom: pure PVA | 3.02 + 7.19 = 10.21 | 34.91 |

Based on the measured sample dimensions, the geometrical areas and exposed-boundary parameters were calculated and are listed in **TableS3**. For cylindrical samples, the projected area, side area and exposed evaporative boundary were calculated from the measured dimensions:

$$A_{proj}=\frac{\pi d^{2}}{4}$$

(S1)

$$A_{side}=\pi dL$$

(S2)

$$A_{evap}=A_{proj}+A_{side}$$

(S3)

$$\Gamma_{A}=\frac{A_{evap}}{A_{proj}}$$

(S4)

The boundary-normalization reduction was calculated as:

$$\phi_{A}=\left( 1 - \frac{J_{exp}}{J_{proj}} \right)\times100$$

(S5)

where $J_{proj}$is the projected-area-normalised evaporation flux and $J_{exp}$is the exposed-boundary-normalised evaporation flux.

**Table S3. Geometrical area and exposed-boundary parameters calculated from measured sample dimensions.**

| **Sample** | $\boldsymbol{A}_{\boldsymbol{proj}}$**/ mm²** | $\boldsymbol{A}_{\boldsymbol{side}}$**/ mm²** | $\boldsymbol{A}_{\boldsymbol{evap}}$**/ mm²** | $\boldsymbol{\Gamma}_{\boldsymbol{A,geom}}$ | $\boldsymbol{\phi}_{\boldsymbol{A,geom}}$**/ %** |
| --- | --- | --- | --- | --- | --- |
| LEB-PVA | 2100.58 | 461.78 | 2562.36 | 1.22 | 18.0 |
| LEB-G0.50 | 1767.05 | 680.74 | 2447.79 | 1.39 | 27.8 |
| HEB-PVA | 784.15 | 1117.95 | 1902.10 | 2.43 | 58.8 |
| HEB-G0.25 | 920.94 | 1001.30 | 1922.24 | 2.09 | 52.1 |
| HEB-G0.50 | 902.95 | 956.82 | 1859.77 | 2.06 | 51.4 |
| HEB-G5.00 | 933.56 | 1085.57 | 2019.12 | 2.16 | 53.8 |
| LEB-BL1 | 1627.61 | 684.78 | 2312.40 | 1.42 | 29.6 |
| LEB-BL2 | 1088.90 | 589.95 | 1678.85 | 1.54 | 35.1 |
| HEB-BL | 957.08 | 1119.86 | 2076.93 | 2.17 | 53.9 |

For LEB-BL1, the geometry-derived $\phi_{A,geom}$is 29.6%, whereas the flux-ratio-derived value used in the main analysis is 27.0%. The performance tables use the flux-ratio-derived value because it is calculated from the reported $J_{proj}$and $J_{exp}$values.

# S3. Material characterization

Surface topography and morphology were characterized using a field-emission scanning electron microscope (Leo Supra 35VP Field Emission Scanning Electron Microscope) and an optical microscope (Nikon LV100ND). Static water contact angle measurements were performed using a drop shape analyzer (DSA10-MK2, KRUSS, Germany). Transient contact-angle measurements were recorded by integrating a high-speed camera (Photron Mini AX100, Photron, Japan) operating at 50 fps with the drop shape analyzer. Sample thickness was determined using a thickness gauge (Mitutoyo, Model 543-394B). Fourier-transform infrared spectra were acquired using an IS10 FT-IR spectrometer to investigate crosslinking characteristics.

Thermal decomposition behavior was evaluated by thermogravimetric analysis (TGA; Mettler Toledo TR, Model TGA/DSC3+) under a controlled temperature ramp up to 1000 °C in an N_2_ atmosphere. The structural characteristics of GNPs within the membranes were examined using Raman spectroscopy (Renishaw inVia Reflex Raman Microscopy System) with a 532 nm laser at room temperature over 300–3500 cm⁻¹. The Raman results were further supported by X-ray diffraction using a Bruker D2 Phaser Desktop diffractometer equipped with a Cu_Kα_ radiation source. Optical absorption was measured using a UV–Vis–NIR spectrophotometer (Agilent Cary 5000).

# S4. Structure and morphology

The morphological characteristics of the fabricated samples are presented in **Figure S1**. The SEM images show the formation of highly porous and interconnected structures generated through freeze-drying, where ice crystal sublimation produced vertically aligned channels that facilitate water transport throughout the sample thickness. The smooth and continuous channel walls indicate that the selected PVA concentration maintained structural integrity during freezing and drying.

The comparison between neat PVA, PVA/0.50% GNP and PVA/5.00% GNP confirms successful GNP incorporation within the PVA matrix. The PVA/0.50% GNP sample shows relatively uniform GNP dispersion without significant structural disruption. In contrast, PVA/5.00% GNP shows stronger nanoparticle agglomeration, suggesting that this loading approaches the upper dispersion limit under the present fabrication conditions. Excessive filler loading can promote local aggregation through increased van der Waals interactions between graphene sheets, which may affect structural uniformity and transport performance.

Image analysis using ImageJ showed that porosity remained high for all samples. The measured porosities were 92.17% for neat PVA, 92.08% for PVA/0.50% GNP and 90.56% for PVA/5.00% GNP. The average pore diameter decreased from 23.78 μm for neat PVA to 16.38 μm for PVA/0.50% GNP and 6.25 μm for PVA/5.00% GNP. This reduction is attributed to the nucleation effect of GNPs during freezing. The increased number of nucleation sites promotes the formation of smaller ice crystals, leading to finer pores after sublimation. The high aspect ratio of GNPs may also restrict unrestricted ice-crystal growth and suppress the formation of large continuous pores.

The optical microscopy image of HEB-BL confirms strong interfacial integration between the neat PVA support and the PVA/0.50% GNP photothermal layer, with no visible gaps or delamination. This continuous interface supports water transport across the bilayer structure. UV–Vis–NIR absorption spectra (**Figure S2a**) show that increasing GNP loading progressively enhances broadband optical absorption, particularly in the visible and near-infrared regions, with the PVA/5.00 wt.% GNP sample exhibiting the highest absorption across the measured wavelength range. This enhanced light harvesting is consistent with the stronger dry photothermal response observed for the GNP-containing evaporators. Transient contact-angle measurements (**Figure S2b**) show that the initial contact angles ranged from approximately 27° for neat PVA to 40° for PVA/5.00 wt.% GNP. The contact angle decreased rapidly after droplet deposition, and complete droplet absorption occurred within approximately 0.4–1.2 s for all freeze-dried PVA-based evaporators. Although increasing GNP loading slightly prolonged the absorption process, the interconnected porous structure produced by freeze-drying enabled rapid capillary infiltration, demonstrating that GNP incorporation did not compromise the hydrophilic liquid-transport characteristics of the PVA matrix.

# S5. Crosslinking, thermal stability and graphitic structure

The FTIR spectra confirm successful crosslinking of the PVA network, evidenced by the broadening of the O–H stretching band (3200–3500 cm^-1^) and changes in the C–O/C–O–C vibration regions (1000–1150 cm^-1^) [5], indicating enhanced intermolecular hydrogen bonding and network formation (**Figure S3a**).

The Raman spectra in **Figure S3b** confirm the incorporation of GNPs within the PVA matrix. Neat PVA showed broad and weak Raman features, consistent with its largely amorphous structure. GNP-containing samples exhibited characteristic D and G bands at approximately 1345 and 1590 cm⁻¹, corresponding to defect-induced vibrations and graphitic sp²-carbon domains, respectively [5, 6]. Pure GNP showed an $I_{D}/I_{G}$ratio of approximately 0.56. After incorporation into the PVA matrix and thermal crosslinking, the ratio decreased slightly to approximately 0.53 for PVA/0.50% GNP and 0.41 for PVA/5.00% GNP. This indicates that the thermal crosslinking treatment did not damage the graphitic structure of the GNPs and that graphitic domains were retained within the porous PVA network.

The XRD patterns in **Figure S3c** further support these findings. Crosslinked PVA exhibited a characteristic broad diffraction peak at approximately 2θ = 19.5°, associated with the semi-crystalline (101) plane of PVA. After GNP incorporation, an additional peak appeared at approximately 2θ = 26°, corresponding to the graphitic (002) plane, which closely matched the XRD pattern of pure GNP. The sharp and well-defined nature of this peak indicates that the GNPs retained a high degree of graphitic ordering without significant structural degradation during dispersion, freeze-drying, or thermal crosslinking. These results are in good agreement with the Raman analysis and further confirm the successful incorporation and structural preservation of GNPs within the porous crosslinked PVA network [7].

The TGA results in **Figure S3d** show that crosslinking and GNP incorporation improve the thermal stability of the freeze-dried PVA membranes. Uncrosslinked PVA powder exhibited rapid thermal degradation between approximately 250 and 450 °C and left 0.51 wt.% residue at 1000 °C. Crosslinked PVA showed a higher residual mass of 2.52 wt.%, indicating that the crosslinked network restricted rapid polymer-chain decomposition.

The PVA/GNP composites showed substantially higher residual mass. PVA/0.50% GNP retained 33.57 wt.% residue at 1000 °C, while PVA/5.00% GNP retained 72.44 wt.%. Pure GNP retained 90.92 wt.% of its initial mass. These results confirm the stabilizing effect of GNP incorporation. All fabricated samples remained thermally stable within the typical operating temperature range of solar-driven interfacial evaporation systems.

# S6. Photothermal and evaporation testing

The dry and wet photothermal responses were measured under one-sun illumination using a SciSun-300-AM1.5G Class AAA solar simulator. Surface temperatures of the exposed top surface were recorded using a FLIR A6751 thermal camera, while evaporation-induced mass loss was measured using an analytical balance (KERN EW 420-3NM, resolution ±0.001 g). The photothermal measurement protocol was developed and optimized based on our previous studies and experimental experience with solar-driven interfacial evaporation systems. All temperature measurements were performed using the same experimental setup, sample positioning, irradiation conditions, and infrared camera configuration to ensure repeatability and enable direct comparison between different evaporator architectures.

For the dry photothermal measurements, the samples were positioned on the same polystyrene (PS) foam support and cotton-wick platform used for the evaporation experiments, with the only difference being that the water reservoir remained empty. This ensured identical thermal boundary conditions between the dry and wet measurements. To minimize the influence of ambient thermal fluctuations, additional PS foam panels were positioned around the experimental setup at a distance of approximately 15 cm from each side.

The relatively high dry-state temperatures arise from the combined effect of the strong broadband light absorption of the graphene nanoplatelets (GNPs) and the highly porous freeze-dried aerogel structure, which promotes heat localization under dry conditions where latent heat removal is absent.

Evaporation tests were performed using DI water, 3.5 wt.% NaCl and 20 wt.% NaCl. The experimental setup consisted of a beaker containing the water reservoir, a polystyrene foam layer as a thermal insulation platform, and cotton wicks to establish continuous capillary water transport from the bulk liquid to the membrane surface. Openings corresponding to approximately 10% of the total foam surface area were introduced into the foam support, and the cotton wicks were uniformly distributed to facilitate water supply. The samples were positioned on the foam-supported reservoir and exposed to one-sun irradiation while the mass loss was continuously monitored.

Control experiments using only the polystyrene foam and cotton-wick support were performed under identical conditions. The support-only evaporation rate was 0.205 kg m^-2^ h^-1^, confirming that the measured evaporation enhancement was dominated by the fabricated evaporators rather than the supporting structure.

Evaporation rates were calculated using two area definitions **(Table S4).** The first uses the projected top surface area, $A_{proj}$. The second uses the combined exposed evaporative boundary, $A_{evap}=A_{top}+A_{side}$. This distinction is necessary because the high-exposed-boundary samples contain additional non-projected evaporation surfaces.

**Table S4. First-hour evaporation rates under DI water, 3.5 wt.% NaCl and 20 wt.% NaCl calculated using projected top area and combined top + side-boundary area. Units: kg m^-2^ h^-1^.**

| **Sample** | **DI water, top** | **DI water, top + side** | **3.5 wt.% NaCl, top** | **3.5 wt.% NaCl, top + side** | **20 wt.% NaCl, top** | **20 wt.% NaCl, top + side** |
| --- | --- | --- | --- | --- | --- | --- |
| LEB-PVA | 0.907 | 0.743 | 0.693 | 0.568 | 0.782 | 0.641 |
| LEB-G0.50 | 1.972 | 1.423 | 1.877 | 1.355 | 1.759 | 1.270 |
| HEB-PVA | 2.299 | 0.948 | 2.117 | 0.873 | 2.143 | 0.884 |
| HEB-G0.25 | 3.181 | 1.524 | 3.071 | 1.471 | 2.969 | 1.422 |
| HEB-G0.50 | 3.059 | 1.486 | 3.059 | 1.486 | 3.175 | 1.542 |
| HEB-G5.00 | 3.233 | 1.495 | 3.008 | 1.391 | 3.217 | 1.488 |
| LEB-BL1 | 1.524 | 1.112 | 1.513 | 1.104 | 1.427 | 1.041 |
| LEB-BL2 | 1.554 | 1.116 | 2.686 | 1.743 | 2.674 | 1.735 |
| HEB-BL | 3.094 | 1.426 | 3.033 | 1.398 | 3.248 | 1.497 |

# S7. Dry photothermal, brine thermal and flux-stability descriptors

Surface temperature was measured using an infrared camera (FLIR A6751) positioned approximately 20 cm from the sample at a viewing angle of 60° from the surface normal. An apparent emissivity setting of 0.95 was used for all measurements. Temperatures were extracted as the maximum temperature within a fixed top-surface region of interest on the illuminated photothermal region. Solar irradiance was calibrated to 1.00 kW m^-2^at the sample position. The sample support, camera position, viewing geometry, illumination configuration, and temperature-extraction procedure were maintained consistently across all experiments. Ambient temperature, relative humidity, and airflow were monitored, and no forced airflow was applied.

The IR temperature-measurement protocol was checked using representative evaporator samples equilibrated in a temperature-controlled oven over the range of 40–200 °C. After thermal equilibration, the samples were measured using the same IR-camera setting, viewing geometry, distance, and region-of-interest procedure used in the solar-evaporation experiments. The maximum deviation between the oven-set temperature and the IR-measured surface temperature was 1°C over the tested range. This check confirms the consistency of the IR measurement protocol for the reported local surface temperatures. The dry photothermal response was characterized by the maximum dry surface temperature, $T_{dry,max}$. Brine stability was evaluated using the late-stage thermal drift, $\Delta T_{late,4h}=T_{4h}-T_{1h}$, the flux-retention ratio, $R_{4h/peak}=J_{4h}/J_{peak}$, and the transport-stability index:

$$\Psi_{4h}=\frac{\Delta T_{late,4h}/(T_{1h}-T_{\infty})}{R_{4h/peak}}$$

(S6)

where $T_{\infty}$is the ambient temperature. The calculated dry photothermal and brine-stability descriptors for all evaporator architectures under 20 wt.% NaCl are presented in **Table S5.**

**Table S5. Dry photothermal and brine-stability descriptors under 20 wt.% NaCl.**

| **Sample** | $\boldsymbol{T}_{\boldsymbol{dry,max}}$**/ °C** | $\boldsymbol{T}_{\boldsymbol{1}\boldsymbol{h}}$**/ °C** | $\boldsymbol{T}_{\boldsymbol{4}\boldsymbol{h}}$**/ °C** | $\boldsymbol{\Delta}\boldsymbol{T}_{\boldsymbol{late,4}\boldsymbol{h}}$**/ °C** | $\boldsymbol{R}_{\boldsymbol{4}\boldsymbol{h}\mathbf{/}\boldsymbol{peak}}$**/ %** | $\boldsymbol{\Psi}_{\boldsymbol{4}\boldsymbol{h}}$ |
| --- | --- | --- | --- | --- | --- | --- |
| LEB-PVA | 77.44 | 47.65 | 59.11 | 11.46 | 90.6 | 0.56 |
| LEB-G0.50 | 163.88 | 67.66 | 131.34 | 63.68 | 66.0 | 2.26 |
| HEB-PVA | 88.68 | 48.99 | 60.25 | 11.26 | 89.4 | 0.53 |
| HEB-G0.25 | 154.08 | 58.35 | 80.02 | 21.67 | 64.7 | 1.00 |
| HEB-G0.50 | 165.28 | 56.91 | 62.25 | 5.34 | 90.9 | 0.18 |
| HEB-G5.00 | 177.36 | 56.04 | 74.42 | 18.38 | 75.0 | 0.79 |
| LEB-BL1 | 157.98 | 64.59 | 89.56 | 24.97 | 63.0 | 1.00 |
| LEB-BL2 | 167.04 | 59.16 | 86.78 | 27.62 | 64.0 | 1.26 |
| HEB-BL | 157.52 | 58.38 | 86.33 | 27.95 | 59.5 | 1.41 |

# S8. Full-duration brine evaporation and boundary normalization

Full-duration brine evaporation rates were calculated from the 20 wt.% NaCl tests using the projected top area and the exposed top + side-boundary area (**Table S6**). These values support the boundary-normalized analysis in the main manuscript.

The boundary-normalization reduction is:

$$\phi_{A}=\left( 1 - \frac{J_{exp}}{J_{proj}} \right)\times100$$

(S10)

The energy consistency index is:

$$\Pi_{E}=\frac{Jh_{fg}}{I}$$

(S11)

using $h_{fg}=2.4\times{10}^{6}$J kg⁻¹ and $I=1000$W m⁻².

**Table S6. Full-duration brine evaporation rates, boundary-normalization correction and energy consistency under 20 wt.% NaCl.**

| **Sample** | $\boldsymbol{J}_{\boldsymbol{proj}}$**/ kg m⁻² h⁻¹** | $\boldsymbol{J}_{\boldsymbol{exp}}$**/ kg m⁻² h⁻¹** | $\boldsymbol{J}_{\boldsymbol{proj}}\mathbf{/}\boldsymbol{J}_{\boldsymbol{exp}}$ | $\boldsymbol{\phi}_{\boldsymbol{A}}$**/ %** | $\boldsymbol{\Pi}_{\boldsymbol{E,proj}}$ | $\boldsymbol{\Pi}_{\boldsymbol{E,exp}}$ |
| --- | --- | --- | --- | --- | --- | --- |
| LEB-PVA | 0.817 | 0.670 | 1.22 | 18.0 | 0.545 | 0.447 |
| LEB-G0.50 | 1.476 | 1.066 | 1.38 | 27.8 | 0.984 | 0.711 |
| HEB-PVA | 2.239 | 0.923 | 2.43 | 58.8 | 1.493 | 0.615 |
| HEB-G0.25 | 2.841 | 1.361 | 2.09 | 52.1 | 1.894 | 0.907 |
| HEB-G0.50 | 3.286 | 1.596 | 2.06 | 51.4 | 2.191 | 1.064 |
| HEB-G5.00 | 3.669 | 1.697 | 2.16 | 53.7 | 2.446 | 1.131 |
| LEB-BL1 | 1.220 | 0.891 | 1.37 | 27.0 | 0.813 | 0.594 |
| LEB-BL2 | 2.436 | 1.581 | 1.54 | 35.1 | 1.624 | 1.054 |
| HEB-BL | 2.847 | 1.312 | 2.17 | 53.9 | 1.898 | 0.875 |

The boundary-normalisation correction, $\phi_{A}$, represents the percentage reduction in the reported evaporation flux when the same mass-loss rate is normalised by the exposed evaporative boundary rather than the projected illuminated area. It should not be interpreted as a reduction in actual mass loss. It reflects the effect of the selected area basis on reported flux.

# S9. Geometry-derived salt-transport path descriptors

The physical salt-loading balance in the main manuscript requires $D_{eff}$, $A_{liq}$, $L_{s}$, $A_{evap}$and $J$. The current dataset allows geometry-derived first-order descriptors to be calculated from the measured sample dimensions (**Table S7**). In this calculation, $A_{liq,geom}$is approximated by the projected area and $L_{s,geom}$is approximated by the measured sample thickness:

$$A_{liq,geom}=A_{proj}$$

(S12)

$$L_{s,geom}=L$$

(S13)

$$\frac{L_{s}}{A_{liq}}=\frac{L}{A_{proj}}$$

(S14)

This table does not replace independent $D_{eff}$measurement. It only provides a geometry-based comparison of salt-transport path length relative to projected transport area.

**Table S7. Geometry-derived salt-transport path descriptors.**

| **Sample** | $\boldsymbol{A}_{\boldsymbol{liq,geom}}\boldsymbol{=}\boldsymbol{A}_{\boldsymbol{proj}}$**/ mm^2^** | $\boldsymbol{L}_{\boldsymbol{s,geom}}$**/ mm** | $\boldsymbol{L}_{\boldsymbol{s}}\mathbf{/}\boldsymbol{A}_{\boldsymbol{liq}}$**/ mm^-1^** | **Geometry-based interpretation** |
| --- | --- | --- | --- | --- |
| LEB-PVA | 2100.58 | 2.84 | 0.00135 | Short transport length; low photothermal activity |
| LEB-G0.50 | 1767.05 | 4.57 | 0.00259 | Short-to-moderate path; high heat loading can dominate |
| HEB-PVA | 784.15 | 11.26 | 0.01436 | Long transport path; low photothermal activity |
| HEB-G0.25 | 920.94 | 9.31 | 0.01011 | Long path with strong photothermal response |
| HEB-G0.50 | 902.95 | 8.98 | 0.00995 | Long path but best thermal/flux stability in measured brine response |
| HEB-G5.00 | 933.56 | 10.02 | 0.01073 | Long path with excessive heat loading |
| LEB-BL1 | 1627.61 | 4.79 | 0.00294 | Bilayer heat localization; moderate path |
| LEB-BL2 | 1088.90 | 5.04 | 0.00463 | Bilayer heat localization; higher path ratio than LEB-BL |
| HEB-BL | 957.08 | 10.21 | 0.01067 | Long path with strong geometry correction |

# S10. Characteristics of Upcycled G3 GNPs

The G3 GNPs used in this study were supplied by Levidian and produced using the LOOP technology, which employs a microwave plasma process to convert methane into graphene and hydrogen. This approach not only enables the production of high-quality graphene materials but also contributes to the sustainability of the process. The physicochemical properties of the upcycled G3 GNPs are summarized in **Table S8**, while the corresponding morphological and structural characteristics are presented in **Figure S6**.

**Table S8. Physicochemical properties of the upcycled G3 GNPs.**

| **Property** | **Value / Description** |
| --- | --- |
| Material Classification | Non-functionalized, few-layer graphene nanoplatelets |
| Elemental Composition | C > 99.9%, O < 0.1% |
| Bulk Density | 60 ± 5 g L^-1^ |
| Agglomerated Particle Size | Average: 23 μm |
| Primary Particle Size | Average: 100nm |
| Primary Particle Thickness | Average: 5.44 nm |
| Number of Graphene Layers | Average: 16 layers |
| Specific Surface Area (SSA) | 120 ± 20 m^2^ g^-1^ (measured by BET) |
| Interlayer Spacing | 0.345 nm (Measured by XRD) |

# S11. Supplementary figures

|  | | |
| --- | --- | --- |
| **(a)** | | |
|  |  |  |
| **(b)** | **(c)** | **(d)** |
|  |  |  |
| **(e)** | **(f)** | **(g)** |
|  |  |  |
| **(h)** | **(i)** | **(j)** |

**Figure S1. Macroscopic morphology, optical microscopy and SEM characterization of the fabricated evaporators.** (a) Macroscopic photographs of the full evaporator library, Optical microscopy images show the overall porous morphology and bilayer interface. SEM images of neat PVA (LEB-PVA, HEB-PVA, and the PVA layer of LEB-BL1, LEB-BL2, and HEB-BL) at (b) 500×, (c) 5k×, and (d) 50k× magnification. (e) PVA/0.50 wt.% GNP (LEB-G0.50, HEB-G0.50, and the PVA/0.50 wt.% GNP layer of LEB-BL1, LEB-BL2, and HEB-BL) at 5k× magnification. (f) PVA/5.00 wt.% GNP (HEB-G5.00) at 5k× magnification. (g) An optical microscopy image of the HEB-BL interface between the PVA/0.50 wt.% GNP top layer and the pure PVA bottom layer. Representative optical microscopy images of (h) neat PVA, (i) PVA/0.50 wt.% GNP, and (j) PVA/5.00 wt.% GNP.

|  |  |
| --- | --- |
| **(a)** | **(b)** |

**Figure S2. Broadband optical absorption of neat PVA, PVA/GNP composites and pure GNP powder. (a)** The spectra show the influence of GNP incorporation on light absorption across the UV–Vis–NIR region. (b) Water uptake and transient wetting behavior of the fabricated evaporators. Time-resolved optical images and transient contact-angle measurements showing rapid droplet spreading and absorption into the porous PVA-based scaffold. The droplets were absorbed within approximately 1.2 s, confirming fast capillary wetting and hydrophilic transport pathways.

|  |  |
| --- | --- |
| **(a)** | **(b)** |
|  |  |
| **(c)** | **(d)** |

**Figure S3. Thermal, structural, and spectroscopic characterization of the fabricated samples**. (a) FTIR spectra of non-crosslinked and crosslinked PVA, showing changes in the O–H and C–O/C–O–C regions after crosslinking. (b) Raman spectra of PVA and PVA/GNP composite membranes, showing the influence of GNP incorporation and thermal crosslinking on graphitic structure and defect density. (c) XRD patterns of pure GNP, crosslinked PVA, and crosslinked PVA/GNP composites. (d) TGA profiles of PVA-based samples with different GNP contents, showing enhanced thermal stability and residual mass after crosslinking and GNP incorporation.

**Figure S4. Schematic of the experimental evaporation setup**.

| **Sample name** | **1 h** | **2 h** | **3 h** | **4 h** |
| --- | --- | --- | --- | --- |
| LEB-PVA   |  |  |  |    |
|  |  |  |  |  |
| LEB-G0.50   |  |  |  |    |
|  |  |  |  |  |
| HEB-PVA   |  |  |  |    |
|  |  |  |  |  |
| HEB-G0.25   |  |  |  |    |
|  |  |  |  |  |
| HEB-G0.50   |  |  |  |  |
|  |  |  |  |  |
| HEB-G5.00   |  |  |  |  |
|  |  |  |  |  |
| LEB-BL1   | NA | NA |  |  |
|  |  |  |  |  |
| LEB-BL2   |  |  |  |    |
|  |  |  |  |  |
| HEB-BL   |  |  |  |    |
|  |  |  |  |  |

**Figure S5. Time-resolved infrared thermograms and optical photographs under 20 wt.% NaCl.** Infrared and optical images of all evaporator architectures during operation with 20 wt.% NaCl at 1, 2, 3, and 4 h. The images show the development of architecture-dependent late-stage thermal drift and delayed secondary-boundary activation. Optical photographs are used to confirm whether visible salt crystallization appears during the same period.

**Figure S6. Morphological and structural characterization of the upcycled G3 GNPs produced via Levidian LOOP technology and provided by the manufacturer.** (a) SEM characterization, (b) TEM characterization, (c) XRD spectrum, (d) Raman spectrum, (e) XPS survey scan, and (f) C1s peak.

# References

[1] Khoei, J. K., et al. Upcycled graphene integrated fiber-based photothermal hybrid nanocomposites for solar-driven interfacial water evaporation. *Desalination* 2023, *562*, 116707.

[2] Sorayani Bafqi, M. S., et al. Expected lifetime of fibrous nanogenerator exposed to cyclic compressive pressure. *Journal of Industrial Textiles* 2022, *51*(2_suppl), 4493S–4505S.

[3] Sun, M., et al. Reconfigurable magnetic slime robot: deformation, adaptability, and multifunction. *Advanced Functional Materials* 2022, *32*, 2112508.

[4] Gohil, J.; Bhattacharya, A.; Ray, P. Studies on the crosslinking of poly(vinyl alcohol). *Journal of Polymer Research* 2006, *13*, 161–169.

[5] Niewiadomski, K., et al., Comparative analysis of crosslinking methods and their impact on the physicochemical properties of SA/PVA hydrogels. Materials, 2024. 17(8): p. 1816.

[6] Dangi, S., et al., Exploration of spectroscopic, surface morphological, structural, electrical, optical and mechanical properties of biocompatible PVA-GO PNCs. Diamond and Related Materials, 2022. 127: p. 109158.

[7] Bafqi, M. S. S., et al. Development of anisotropic nanofibrous hybrid membranes coated with upcycled graphene for enhanced adsorption of emerging contaminants from drinking water. *Environmental Research* 2025, *282*, 122086.

[8] Saleh, A., et al., Comprehensive examination of synthesis, microstructure, and radiation shielding effectiveness of multi-layered polymeric GNP-nanocomposites. Radiation Physics and Chemistry, 2025. 237: p. 113078.
